# Imidazolium-modification enhances photocatalytic CO_2_ reduction on ZnSe quantum dots†

**DOI:** 10.1039/d1sc01310f

**Published:** 2021-05-17

**Authors:** Constantin D. Sahm, Eric Mates-Torres, Nora Eliasson, Kamil Sokołowski, Andreas Wagner, Kristian E. Dalle, Zehuan Huang, Oren A. Scherman, Leif Hammarström, Max García-Melchor, Erwin Reisner

**Affiliations:** Yusuf Hamied Department of Chemistry, University of Cambridge Lensfield Rd Cambridge CB2 1EW UK reisner@ch.cam.ac.uk http://www-reisner.ch.cam.ac.uk; School of Chemistry, CRANN and AMBER Research Centres, Trinity College Dublin, College Green Dublin 2 Ireland garciamm@tcd.ie; Department of Chemistry, Ångström Laboratory, Uppsala University Box 523 751 20 Uppsala Sweden leif.hammarstrom@kemi.uu.se; Melville Laboratory for Polymer Synthesis, Yusuf Hamied Department of Chemistry, University of Cambridge Lensfield Rd Cambridge CB2 1EW UK; Institute of Physical Chemistry, Polish Academy of Sciences Kasprzaka 44/52 01-224 Warsaw Poland

## Abstract

Colloidal photocatalysts can utilize solar light for the conversion of CO_2_ to carbon-based fuels, but controlling the product selectivity for CO_2_ reduction remains challenging, in particular in aqueous solution. Here, we present an organic surface modification strategy to tune the product selectivity of colloidal ZnSe quantum dots (QDs) towards photocatalytic CO_2_ reduction even in the absence of transition metal co-catalysts. Besides H_2_, imidazolium-modified ZnSe QDs evolve up to 2.4 mmol_CO_ g_ZnSe_^−1^ (TON_QD_ > 370) after 10 h of visible light irradiation (AM 1.5G, *λ* > 400 nm) in aqueous ascorbate solution with a CO-selectivity of up to 20%. This represents a four-fold increase in CO-formation yield and 13-fold increase in CO-selectivity compared to non-functionalized ZnSe QDs. The binding of the thiolated imidazolium ligand to the QD surface is characterized quantitatively using ^1^H-NMR spectroscopy and isothermal titration calorimetry, revealing that a subset of 12 to 17 ligands interacts strongly with the QDs. Transient absorption spectroscopy reveals an influence of the ligand on the intrinsic charge carrier dynamics through passivating Zn surface sites. Density functional theory calculations indicate that the imidazolium capping ligand plays a key role in stabilizing the surface-bound *CO_2_^−^ intermediate, increasing the yield and selectivity toward CO production. Overall, this work unveils a powerful tool of using organic capping ligands to modify the chemical environment on colloids, thus enabling control over the product selectivity within photocatalyzed CO_2_ reduction.

## Abstract 2

A photocatalyst system consisting of ZnSe quantum dots modified with a thiolated imidazolium capping ligand for visible light-driven reduction of aqueous CO_2_ to CO is reported without the need for a metal complex co-catalyst.

## Graphical abstract

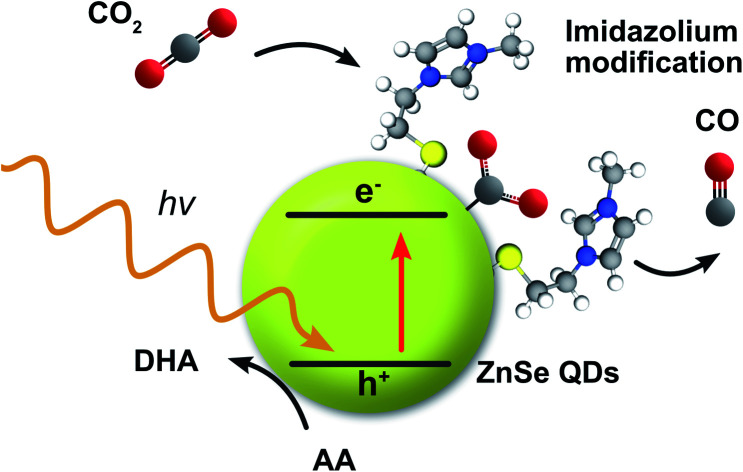

## Introduction

The sustainable generation of carbon neutral fuels is expected to play a critical role in the future energy supply. Particularly, artificial photosynthesis is a process that aims to convert CO_2_ and water into chemical fuels using sunlight for the development of a closed, CO_2_-neutral carbon cycle.^1,2^ Semiconducting nanoparticles, such as quantum dots (QDs), are suitable light absorbers for artificial photosynthesis due to their high surface area and dispersibility,^3^ unique photophysics^4^ and tunable surface chemistry.^5^ In particular, colloidal chalcogenide QDs are established photocatalysts for the H_2_ evolution reaction (HER)^4,6–8^ and have also been reported for CO_2_ reduction by combining QDs with molecular transition metal CO_2_ reduction co-catalysts.^9,10^ Yet, examples of photocatalytic systems that operate in the absence of an additional co-catalyst are scarce and only a few approaches have recently emerged in order to render QDs active for CO_2_ reduction through doping,^11^ or surface enrichment with Cd-containing catalytic sites.^12^

The local chemical environment of CO_2_ reduction active sites is considered a key determinant in the design of efficient catalysts.^13^ Imidazolium based ionic liquids (ILs) have been explored in numerous reports due to their influence on CO_2_ electroreduction although the mechanistic details still remain controversial.^14–16^ In electrochemical CO_2_ reduction, early studies reported that the additive 1-ethyl-3-methylimidazolium tetrafluoroborate (EMIM-BF_4_) in the electrolyte solution (18 mol%) with a Ag electrode stabilizes the *CO_2_^−^ intermediate (where * denotes a surface active site) through complexation to effectively lower the initial activation barrier.^17^ Follow-up studies have demonstrated the participation of the IL-imidazolium motif in CO_2_ reduction by forming IL–CO_2_ adducts,^14,18^ whereas others showed secondary coordination sphere effects through (coulombic) stabilization,^19^ electric fields,^20^ H-bonding,^21^ attraction of CO_2_ to the catalytic active site^22,23^ or formation of a favorable microenvironment^24^ in close proximity to the active center. The use of imidazolium-ILs in photochemical CO_2_ reduction is far less explored. A tetrabutylphosphonium pyridine-oleate IL was the medium for direct air capture of atmospheric CO_2_ and subsequent photocatalytic conversion on a conjugated polymer.^25^ EMIM-BF_4_ was used in a homogeneous photochemical system containing a Ru dye and Co^II^ salt, but the exact role of the IL remains unclear.^26^

**Fig. 1:**
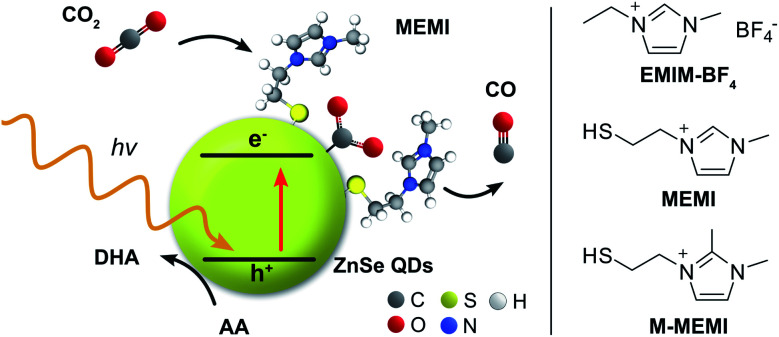
Schematic representation of the photocatalyst system consisting of ligand-free ZnSe–BF_4_ QDs (yellow sphere; BF_4_^−^ anions are omitted for clarity) modified with the capping ligand MEMI or M-MEMI (halide conterion not shown) for visible light-driven CO_2_ to CO reduction in water using ascorbic acid (AA) as the sacrificial electron donor. The related ionic liquid EMIM-BF_4_ (without a thiol surface-anchor) was used for comparison. DHA: dehydroascorbic acid.

Herein, we present a surface modification strategy for ZnSe-QDs, typically highly active towards HER,^27^ which enables photocatalytic CO_2_ to CO reduction even in the absence of a transition metal-based co-catalyst (Fig. 1). We show the modification of the chemical environment of the QDs by designing a capping ligand, which incorporates the imidazolium motif and binds to the QD surface *via* a thiol group. The capping ligand–QD interactions are quantified using ^1^H-NMR spectroscopy, isothermal titration calorimetry (ITC), and electrokinetic *ζ*-potential measurements. The mechanistic insights are provided through transient absorption (TA) spectroscopy and density functional theory (DFT) calculations with a proposal for a QD-surface promoted CO_2_ reduction pathway.

## Results and discussion

### Synthesis of the photocatalyst components

ZnSe QDs were prepared as reported previously^9^ by heating zinc stearate and selenium in octadecene to 300 °C followed by reactive ligand stripping using Me_3_OBF_4_ to remove stearate from the surface and replace it by weakly coordinating BF_4_^−^ anions (ZnSe–BF_4_).^28^ The ZnSe–BF_4_ particles were pseudo-spherical with a diameter of 4.5 ± 0.7 nm as determined by transmission electron microscopy (TEM, Fig. S1A–C†). The UV-vis spectrum shows a visible-light response with a first excitonic absorption maximum at 416 nm (Fig. S1D†). Powder X-ray diffraction indicates a zinc blende crystal structure with broadening of the signals due to nanostructuring (Fig. S1E†). The capping ligand 3-(2-mercaptoethyl)-1-methyl-imidazolium halide (MEMI) was synthesized by reacting 1-methyl-imidazole with 1,2-dibromoethane, followed by substitution of bromide by thioacetate and acid hydrolysis to yield the thiol-modified imidazolium compound MEMI (see ESI† for synthetic details and characterization).

### Ligand–QD interactions

Interactions of the capping ligand MEMI with ligand-free ZnSe–BF_4_ QDs in aqueous solution were first studied by liquid-phase ^1^H-NMR spectroscopy, isothermal titration calorimetry (ITC), and electrokinetic *ζ*-potential measurements. NMR spectroscopy has recently shown to be a useful method to probe interactions of small molecules with the surface of colloidal nanocrystals, providing insights into binding equilibria and allowing distinction between bound and free ligands.^29–31^ Binding is typically indicated by significant broadening of the signals stemming from protons localized in close proximity to nanocrystal interfaces, arising from their slow and nonuniform tumbling.^32,33^

**Fig. 2:**
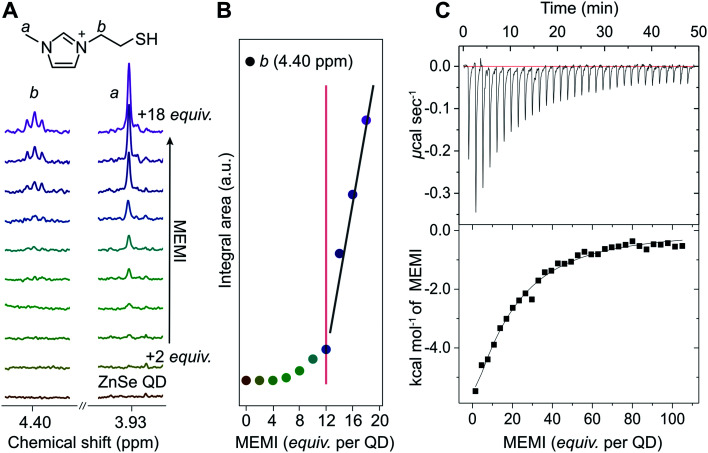
(A) ^1^H-NMR spectroscopy titration experiment with aliquots of MEMI being added to a D_2_O suspension of 5 μM ZnSe–BF_4_ QDs (2 equiv. (mol MEMI per mol QD) per step). Selected signals (4.40 and 3.93 ppm) are shown for clarity. (B) Plot of integral area from NMR experiments *vs* increasing MEMI loading from the NMR experiments. The signals characteristic for MEMI (*i.e.*, 4.4 ppm) start to disappear at <12 equiv. per ZnSe–BF_4_ QD, suggesting that a ZnSe–BF_4_ QD is able to accommodate approximately 12 MEMI molecules. Additional data and the full spectra are shown in the ESI (Fig. S2 and S3†). The fitted lines aim to help guide the eye. (C) ITC curve and plot obtained by titration of MEMI (0.5 mM) into an aqueous ZnSe–BF_4_ QD solution (1 μM).

The affinity of MEMI to the ZnSe–BF_4_ QDs was studied by ^1^H-NMR spectroscopy (D_2_O, 25 °C, Fig. 2A, B, S2 and S3†). In a standard titration experiment, small quantities of MEMI (*i.e.*, 2 equiv. (mol MEMI per mol QD) per injection) were added stepwise to a suspension of ZnSe–BF_4_ QDs. For quantities of MEMI < 12 equiv. per ZnSe–BF_4_ QD, the signals of the ligand essentially vanish, which suggests a strong binding affinity of MEMI to the QD interfaces (Fig. 2A, B). However, when the amount of MEMI is >12 equiv., a linear increase in signal intensity of sharp peaks assigned to freely-diffusing MEMI is observed, indicative of accumulation of MEMI in the bulk solution. These ^1^H-NMR spectra suggest that single ZnSe–BF_4_ QDs are able to accommodate up to 12 MEMI molecules (surface coverage *ca.* 20%, see ESI for details†) tightly bound to the QD interfaces, leaving accessible surface area for other species (including solvated MEMI), which interact in a weaker/dynamic manner.

The strong interaction between MEMI and the QDs was further corroborated by ITC, a quantitative technique for determining thermodynamic parameters of dynamic interactions in solution. Widely used in bio- and supramolecular chemistry,^34,35^ ITC is increasingly being utilized to study interfacial interactions of ligands with colloidal nanoparticles.^36–38^ Titration of MEMI into ZnSe–BF_4_ QDs shows a significant exothermic response (up to −6 kcal mol^−1^) at low ligand concentration (1 μM) that rapidly saturates (Fig. 2C and S4†), indicating a strong affinity of MEMI for the QD surfaces. Through fitting of the ITC data with the *one set of sites* binding model (see Fig. S4 for details†), the binding affinity (*K*_a_) was calculated to be 2.5 × 10^4^ M^−1^, and the number of binding sites (N) was estimated to be 17 ± 5, which is in good agreement with the NMR data and is further corroborated by electrokinetic *ζ*-potential measurements. Addition of positively-charged MEMI to a suspension of ZnSe–BF_4_ QDs changed the electrokinetic *ζ-*potential of the QDs (*ζ* = +45 mV; Fig. S5†) to even more positive values (*ζ* = +55 mV), indicative of the decoration of ZnSe–BF_4_ QD interfaces with the ligand.

### Photocatalytic CO_2_ reduction

The photocatalytic activity of the ZnSe–BF_4_ QDs was studied under a constant flow of CO_2_ and automated in-line gas chromatography (Fig. S6,† detailed description in ESI†). Compared to conventional photocatalysis typically conducted in sealed photoreactors accompanied with the accumulation of gaseous products in the headspace, a continuous-flow setup offers several advantages. Besides the convenience of automated gas sampling using a flow-selection valve equipped gas chromatograph, the continuous-flow setup yields a high resolution with samples injected every *ca.* 4 min as well as a constant removal of reaction products avoiding build-up of excessive pressure and potential catalyst poisoning.

**Fig. 3:**
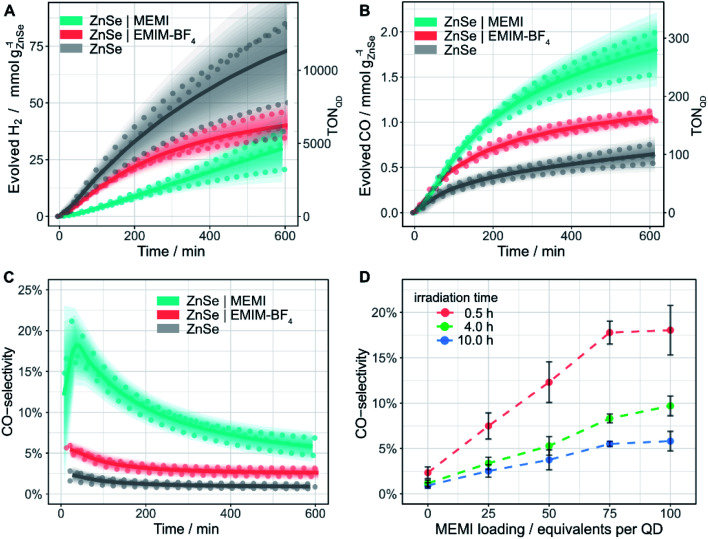
Photocatalytic reduction of aqueous CO_2_ in the presence of ligand-free ZnSe–BF_4_ QDs and modified QDs ZnSe|EMIM-BF_4_ or ZnSe|MEMI: (A) H_2_ and (B) CO evolution and (C) CO-selectivity as a function of irradiation time; 0.5 μM ZnSe–BF_4_, 50 μM EMIM-BF_4_ or MEMI, molar ratio 100 (ligand to QD). (D) CO-selectivity as a function of the MEMI loading for ZnSe|MEMI. Conditions: AM 1.5G, *λ* > 400 nm, 100 mW cm^−2^, 0.5 μM ZnSe–BF_4_, 0.1 M AA, pH 6.5, CO_2_ constant flow (4 sccm), 25 °C. The scatter plots show measured values for a given experimental condition conducted in triplicate. Lines represent the smoothened mean of a triplicate experiment. The shaded area represents the standard deviation where the opacity is proportional to the normal distribution around the mean. For details, see ESI.†

Samples (0.5 μM ZnSe–BF_4_) were irradiated using UV-filtered simulated solar light (*λ* > 400 nm, AM 1.5G, 100 mW cm^−2^) in an aqueous ascorbic acid (AA) solution (3 mL, 0.1 M, pH 6.5) at 25 °C. Besides previously reported HER activity,^27^ non-functionalized ZnSe–BF_4_ also display a marginal activity toward CO evolution (Fig. 3 and Table S1†). After 10 h of continuous irradiation, a total of 0.64 ± 0.11 mmol_CO_ g_ZnSe_^−1^ is produced with a modest CO-selectivity (defined as *n*(CO)/(*n*(CO) + *n*(H_2_))) of <3%. We confirmed the origin of generated CO from reduced CO_2_ by ^13^C-isotopic labelling experiments (Fig. S7†) to exclude any contribution from decomposition of residual organic impurities or solvents.

Encouraged by the ability of the QDs to reduce small amounts of CO_2_ even in the absence of any co-catalyst, we sought to enhance the performance by surface modification with an imidazolium moiety. The addition of 100 equiv. per QD of the freely diffusing IL EMIM-BF_4_ (Fig. 1) to the solution reduces HER activity to about half (Fig. 3A) and enhances CO formation (1.06 ± 0.06 mmol_CO_ g_ZnSe_^−1^ after 10 h irradiation, CO-selectivity < 5%) (Fig. 3B). This observation agrees with the activity-enhancing effect of EMIM-BF_4_ in previously reported electrochemical systems (see above).^17^

Direct *in situ* self-assembly of the imidazolium moiety on the QD surface *via* a thiol anchoring group gives ZnSe|MEMI (100 mol MEMI per mol QD) (Fig. 1). The immobilization of the ligand on the QD further enhances the production of CO to a significant extent (1.78 ± 0.23 mmol_CO_ g_ZnSe_^−1^ after 10 h irradiation, TON_QD_ = 277) at the expense of the HER, leading to a CO-selectivity of up to 18% (Fig. 3). After 10 h irradiation, this represents a 6.4-fold increase in CO selectivity over the non-functionalized ZnSe–BF_4_ (Table S1†). Both product evolution rates (H_2_ and CO) decay over longer irradiation times (Fig. S8 and S9†), which is presumably mainly governed by accumulation of oxidation products (DHA) on the QD surface (as investigated previously^39,40^), aggregation processes and the resultant reduction of the overall surface area, slow degradation of the QDs and/or loss/decomposition of the capping ligand MEMI.

TEM micrographs of the particles after irradiation show QD-aggregates, however, individual particles are still clearly distinguishable with a nanocrystalline morphology (Fig. S10A†). UV-vis spectra of the QDs collected after photocatalysis exhibit increased scattering (presumably due to agglomeration), but the absorption onset remains unchanged compared to a fresh QD solution (Fig. S10B†). These observations corroborate the chemical stability of the particles and suggest that the photocatalytic activity is mainly limited by QD surface changes due to their aggregation processes, accumulation of DHA and/or loss of ligand as the main contributions.

Next, we explored if the amounts of evolved H_2_ and CO could be modulated by varying the loading of MEMI on the QD surface (Fig. S8†). A molar ratio (MEMI to the QD) of 25 significantly enhances the initial CO formation rate over non-functionalized QDs by a factor of two. Higher loadings do not accelerate the CO production rate further, which saturates within the first 100 min of the experiment, regardless of the ligand loading (Fig. S8B and S9†). This observation agrees with ^1^H-NMR spectroscopy titration experiments and ITC measurements, where only a small number of ligands (*ca.* 10–17) strongly interact with the QD surfaces. Nevertheless, higher loadings (molar ratio 50–100) further suppress HER (Fig. S8A†) and maintain enhanced CO formation at longer irradiation times (>200 min) (Fig. S8D,† S9†). Notably, at a given irradiation time, a near-linear correlation between the MEMI loading (between 0 and 75 equiv. per QD) and CO-selectivity is observed, which starts to level off at a high molar ratio of approximately 75 (Fig. 3D). These observations suggest that the strongly interacting MEMI ligands promote CO_2_ reduction while higher loadings further block HER activity through the weak/dynamic interaction of the MEMI ligands with the QDs. The excess ligands may also allow replenishment of decomposed/desorbed ligands after longer irradiation times.

Only traces of formate (<3% of carbonaceous products, Table S3†) are formed and no other CO_2_-reduction products (*e.g.* methane, methanol) are detected. Only negligible amounts of CO are produced under N_2_ flow (Fig. S11†) and no gaseous products are detected in the dark or in the absence of QDs or AA, demonstrating that all components of the photocatalytic system are required (Table S2†). ^13^C-labelling also confirmed CO_2_ as the sole origin of CO in the presence of MEMI (Fig. S7†). To rule out the possibility that the influence of MEMI originates purely from the presence of a thiol group, we conducted a control experiment with a ligand consisting of a thiol with no additional functionality, 1-butanethiol (BuSH), which resulted in a similar product distribution compared to non-functionalized ZnSe (Fig. S12†). This result indicates that the imidazolium functional group on the ligand is essential for the suppressed HER and enhanced CO production relative to bare ZnSe. We next considered the possibility that the thiol group could be oxidized under photocatalytic conditions, which would effectively render it an electron donor, as previously reported.^41,42^ However, ^1^H-NMR spectroscopy of a ZnSe|MEMI solution after 1 h of solar irradiation did not show any signals from a potential disulfide product (Fig. S13†).

It was previously reported that the imidazolium motif in ILs can participate in electrochemical CO_2_ reduction either by *in situ* formation of a carbene^18^ and subsequent attack of CO_2_, or by directly forming a CO_2_–IL adduct after 1e^−^ reduction of the imidazolium ligand.^14^ Both mechanistic pathways involve a carbene intermediate and the imidazolium could essentially be considered a co-catalyst. In order to probe the feasibility of such a pathway for the ZnSe|MEMI system, we prepared an analogue of MEMI which is methyl-protected at the imidazolium C_2_ position (M-MEMI) to effectively inhibit the formation of a carbene. In a photocatalytic comparison experiment, ZnSe|M-MEMI exceeds ZnSe|MEMI in both CO formation and CO-selectivity reaching a benchmark 2.38 ± 0.19 mmol_CO_ g_ZnSe_^−1^ after 10 h irradiation (TON_QD_ (CO) = 370) with an improved selectivity towards CO of 12.0% (Fig. S14 and Table S1†). This observation precludes a solely imidazolium-catalyzed CO_2_ reduction mechanism. In contrast, we propose that the CO_2_ reduction proceeds on the ZnSe surface with increased efficiency by the imidazolium-ligand, which is supported by the ability of QDs to reduce CO_2_ even in the absence of MEMI.

While the CO-selectivity remains relatively low in all cases (<20%), the changes in product selectivity are notable as the CO-selectivity of ZnSe|M-MEMI is enhanced 13-fold compared to non-functionalized ZnSe–BF_4_ under optimized conditions. This underscores the potentially wide-ranging impact of this conceptionally novel ligand modification strategy. The average external quantum efficiency (EQE_CO_) for the best performing system (ZnSe|M-MEMI) was 0.29 ± 0.13% (400 nm monochromatic light, 1.0–1.5 mW cm^−2^, 2 h, Table S4†). Additionally, the rate of CO evolution (238 μmol g_QD_^−1^ h^−1^) is amongst the highest for QD-photocatalyzed CO_2_ to CO reduction in aqueous solution,^43^ with higher activities only being reported in organic solvents using monochromatic blue LEDs as the light source,^12,44^ or in the presence of a transition metal-based molecular electrocatalyst.^9,45^

### Charge carrier dynamics

**Fig. 4:**
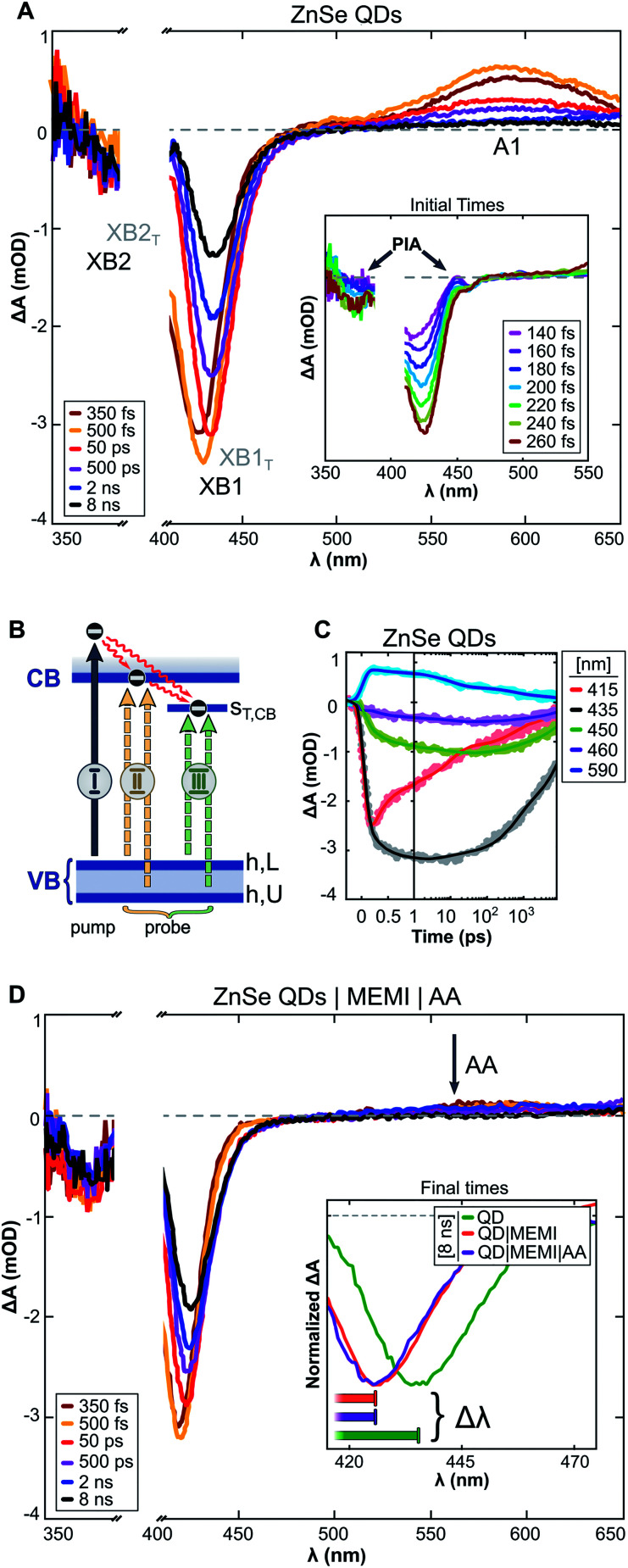
(A) Differential absorbance (Δ*A*) spectra recorded by transient absorption (TA) experiments of aqueous ZnSe-QDs. Pump: 400 nm. Inset: early-time TA spectra. (B) Schematic diagram of optical transitions related to the (I) pump, (II) probe: VB-to-CB excitonic transitions (XB1 and XB2), and (III) probe: VB-to-CB trap transitions (XB1_T_ and XB2_T_). (C) Kinetics of ZnSe QDs extracted from the transient spectra presented in (A). (D) Δ*A* spectra of ZnSe QDs in an aqueous ascorbate (AA, 10 mM, pH: 6.7) solution with 100 equiv. per QD of thiol functionalized MEMI ligand. Inset: 8 ns TA spectra of ZnSe, ZnSe|MEMI and ZnSe|MEMI|AA. The horizontal bars illustrate the magnitude of the dynamic redshift experienced by the bleach bands, ranging from 150 fs (left) to 8 ns (right).

The excited state dynamics of aqueous ZnSe and ZnSe|MEMI (1 : 100) were monitored by transient absorption (TA) spectroscopy experiments. The samples were pumped with 400 nm pulses and probed in the UV-Vis/NIR region at varying pump–probe delays (Δ*t*_p–p_ ≤ 8 ns), see the ESI and Fig. S15† for further details. The transient spectra of the QDs (Fig. 4A) show long-lived (>8 ns) negative bands at 410–450 nm and 365–405 nm, as well as a positive signal (>525 nm) extending into the NIR (Fig. S16A†). Similar features were observed in a previous TA study on these QDs,^9^ apart from the higher energy negative band (denoted XB2) owing to the previous lack of probe coverage in the UV. Herein, derivative-like features are observed in the early-time TA spectra (inset of Fig. 4A), attributed to Coulomb induced biexciton shifts,^46,47^ resulting in photo-induced absorption (PIA) signals at the lower energy sides of the negative signals. The PIA signals decay within a few hundred femtoseconds, indicating carrier relaxation to the band edge states, leaving the optical response to be dominated by state filling effects.^47^

We assign the negative bands after carrier relaxation (Δ*t*_p–p_ ≈ 350 fs) to the bleaching of valence band–conduction band (VB–CB) excitonic transitions (denoted XB1 and XB2), with dynamics that reflect a single band edge population. Specifically, we associate XB1 and XB2 with two distinct transitions (Fig. 4B(II)) that involve the VB edge (VB_h,L_) and a deeper hole state (VB_h,U_) that share a common CB electron state, in accordance with TA studies on the II–VI analogues of ZnSe.^47–49^ At probe delays Δ*t*_p–p_ > 350 fs the maxima of XB1 and XB2 experience a redshift with a concomitant band broadening (brown → yellow → red spectra in Fig. 4A). This alludes to the presence of optically active sub-band trap states, in line with previous studies on similar ZnSe materials.^49–52^

The trapped carrier nature was revealed by introducing AA (10 mM, pH ∼6.7) as a hole scavenger, allowing the decoupling of electron and hole dynamics. A hole-contribution to the positive transient was evidenced by the ultrafast removal of A1 (>525 nm) in the presence of AA (Fig. 4D: ZnSe|MEMI|AA, note that the influence of MEMI is discussed further below). We assign A1 specifically to trapped holes, as reported previously.^9^ The dynamic redshifts of XB1 and XB2, however, are preserved in the presence of AA. Previously, the presence of optically allowed band-to-trap transitions was evidenced by the faster recovery of a distinct sub-band bleach (*λ*_bleach_: 550 nm) upon electron transfer events from the QDs to a co-catalyst.^9^ Herein, the energetically distinct hole states (Fig. 4B) share a common electron trap state (S_T,CB_), seemingly in much closer proximity to the CB edge, which upon CB-to-S_T,CB_ trapping events result in the bleaching of sub-band transitions (XB1_T_ and XB2_T_: III) superimposed on the low-energy side of the VB–CB excitonic resonance (XB1 and XB2: II). The average of electron trapping rates is reflected in the redshift dynamics (Fig. 4C), following the CB-edge state filling sensitive recovery of XB1/XB2 (red trace) and correlated growth of XB1_T_/XB2_T_ (grey-green-violet trace) as population transfer proceeds.

The passivation of unsaturated sites on the QD surface is expected to lower the density of trap states related to surface defects which, in the present system, could manifest as attenuated VB-to-S_T,CB_ transition probability. This is reflected in the TA dynamics as a smaller wavelength shift with time of the bleach maximum (Δ*λ*_bleach_ inset Fig. 4D: ZnSe|MEMI|AA, Fig. S16B and D:† ZnSe|MEMI) from initial to final times (Δ*t*_p–p_: ∼150 fs to 8 ns, horizontal bars) upon the addition of 100 equiv. per QD of the thiol functionalized MEMI ligand compared to the neat QDs (orange/violet *vs.* green spectra) – consistent with fewer CB-to-S_T,CB_ trapping events. The dynamics of the A1 band remains unperturbed upon MEMI binding (see Fig. S16C†), which implies that the ligand does not influence hole-trap states. These observations support a high ZnSe–MEMI binding affinity, and indicate that at least a subset of the ZnSe trap states (S_T,CB_) can be associated with unpassivated surface sites. The CB-related trap states are likely metal cation based,^47,53^ indicating a ZnSe(Zn)–MEMI(thiol) binding site.

Overall, the TA experiments reveal that the MEMI ligand influences the charge carrier dynamics of the QDs, and that this effect most likely is surface-defect related with a ligand binding site corresponding to unpassivated Zn. The high surface-to-volume ratio, resulting from the abrupt terminations of the crystal lattice, leaves ligand-free QDs with a large portion of electronic trap states that can have a detrimental effect on the charge separation ability. Control experiments with BuSH, however, clearly indicate that surface passivation alone is not sufficient to explain the enhanced CO_2_RR activity and selectivity in ZnSe|MEMI compared to the unpassivated QDs. Furthermore, the lack of an observable change in the trapped-hole dynamics in the presence of MEMI, monitored through TA experiments, rules out the possibility that the observed changes in photocatalytic activity from addition of MEMI are due to MEMI affecting the hole dynamics; this is in line with the photocatalytic control experiments above in which MEMI did not act as electron donor. Whether the ability of the unpassivated QDs to reduce CO_2_ results from charge transfer involving the remaining CB population, or whether trapped electrons are accessible to CO_2_ in the present system, remains to be explored, but can be key information in the design of ligands in similar systems where trap states may have an adverse effect on, or promote, charge separation. These experiments point unequivocally towards a more unique role for the imidazole-based ligands, which extends beyond the intrinsic QD charge carrier dynamics into the second-coordination sphere. This assumption is corroborated by the observation that MEMI affects both reaction products differently (suppresses HER, enhances CO), which further affirms an influence beyond the intrinsic photophysics in the particle's chemical environment.

### DFT calculations

Having excluded a mechanism directly catalyzed by the MEMI-ligand, as well as the influence of MEMI on the intrinsic charge carrier dynamics in order to explain the enhanced CO formation activity promoted by MEMI, we next explore secondary-coordination sphere effects of MEMI on QD-surface promoted CO_2_ reduction. The mechanism of CO_2_ reduction has been widely investigated on numerous electrocatalytic materials, but the exact nature of the pathway and intermediates is still under debate.^54,55^ For CO_2_ to CO reduction, the pathway is believed to proceed either *via* an electron transfer (ET) to form a surface stabilized *CO_2_^−^ radical or *via* a concerted proton-coupled electron transfer (PCET) to yield *COOH. This first step is typically the most energy demanding and it is followed by another PCET, abstraction of H_2_O, and the subsequent desorption of *CO.^56^ Hence, unravelling the nature of the first intermediate is essential for the elucidation of the reaction mechanism and rationalization of the catalytic activity.

To assess the catalytic competence of the ZnSe-QDs toward CO_2_ to CO reduction and shed light on the overall mechanism, we sought to conduct a comprehensive computational investigation by means of periodic DFT calculations using the Perdew–Burke–Ernzerhof (PBE) functional with Grimme's D3 dispersion corrections (see ESI for details†). Firstly, the predominant morphology of the ZnSe-QDs was investigated by modelling their equilibrium shape *via* the Wulff construction method. The resulting equilibrium crystal shape consisted of a rhombic dodecahedron exposing exclusively the (220) lattice plane (Fig. S17†), in agreement with previous theoretical works.^57^ The coverage of MEMI ligands on the ZnSe(220) surface was subsequently investigated, ultimately leading to a 50% coverage (relative to the available Zn surface sites) as the most energetically favorable (Fig. S18†).

Once the resting state of the ZnSe|MEMI system was assessed, we set out to investigate the HER activity on both the bare and MEMI-terminated ZnSe(220) surfaces, with and without the presence of a photogenerated electron. After assessing the *H binding energy on all possible active sites in both systems (Fig. S19†), calculations revealed that both the bare ZnSe and ZnSe|MEMI systems can only promote HER atop the Zn surface atoms and in the presence of a photogenerated electron. Hence, we conclude that the enhanced catalytic performance of ZnSe|MEMI QDs in our experiments stems from the stronger binding of the MEMI ligands through the thiol group compared to that of H atoms, which blocks the HER–active Zn surface sites hindering this competing reaction. This finding is in line with the observation from TA spectroscopy that indicate MEMI passivates Zn sites, that were found here responsible for HER.

**Fig. 5:**
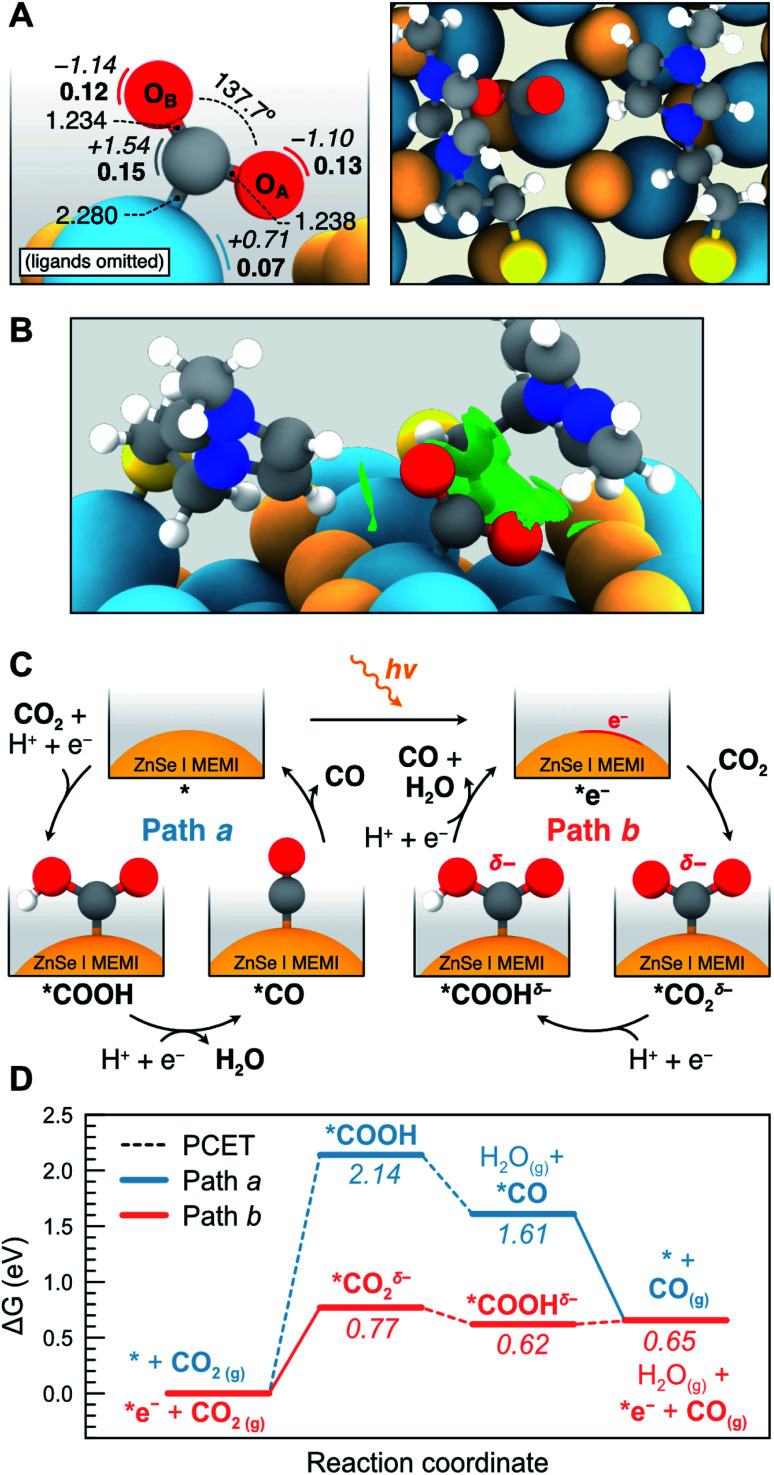
(A) Side (left) and top (right) views of the *CO_2_^*δ*−^ intermediate with the relevant bond distances (in Å) and angles. Calculated atomic Bader charges (in *e*) are displayed in italics beside each atom, while magnetizations (in *μ*_B_) are shown in bold. Note that neighboring MEMI ligands have been omitted in the side view for clarity. Color code: C (grey), O (red), H (white), S (yellow), Zn (teal), Se (orange). (B) Side view representation of the NCI isosurfaces (with an isovalue of 0.35 e^−^ a.u.^−3^) responsible for the stabilization of the *CO_2_^*δ*−^ intermediate. (C) Scheme illustrating the two proposed pathways for the CO_2_ to CO reduction on the ZnSe|MEMI system in the absence (path *a*, in which the reaction begins with a PCET) and presence (path *b*, starting with an ET) of a photogenerated electron. (D) Calculated Gibbs energy diagrams for the CO_2_ to CO reduction *via* path *a* (in blue) and path *b* (in orange) at the experimental conditions (see ESI for details†). Steps involving a PCET are denoted as a dotted line. The structures of all the reaction intermediates are shown in Fig. S21.†

We then turned our attention to the CO_2_ activation on the bare and MEMI-functionalized surfaces, with and without the presence of a photogenerated electron. Importantly, all the attempts to adsorb CO_2_ on the bare ZnSe(220) surface were unsuccessful, resulting in the dissociation of CO_2_ from the surface into the gas phase. A similar result was obtained on the neutral ZnSe|MEMI system, and only when a photogenerated electron was introduced in the simulation, CO_2_ could be stabilized on the Zn surface sites (Fig. 5A), which represent the unique active sites for hydrogen and CO_2_ adsorption, and therefore, HER and CO_2_RR. Further insight was obtained from the calculated magnetic moments and Bader charge analysis on the C and O atoms closest (O_A_) and furthest (O_B_) from the surface, which indicated that the photogenerated electron is delocalized between the adsorbed CO_2_ and the QD surface.

Hence, we conclude that CO_2_ is activated upon interacting with the photogenerated electron on the QD surface, leading to a negative charge density and radical behavior build-up, which we denote as *CO_2_^*δ*−^. We also note that, although *H and *CO_2_^*δ*−^ bind preferentially on the same surface Zn sites, the functionalization of the QD surface with MEMI ligands has an opposite effect on the HER and CO_2_ reduction activity. In particular, the partial coverage and positive charge of MEMI ligands hinder the HER by decreasing the amount of accessible Zn active sites for this reaction and increasing the electrostatic repulsion potential between protons and MEMI, while also enabling the stabilization of the *CO_2_^*δ*−^ intermediate on the surface. Consequently, CO_2_ reduction is promoted, in agreement with experiments from photocatalysis and charge carrier dynamics. However, given the smaller size of the H-atoms compared to CO_2_, CO selectivity is expected to plateau at high concentrations of MEMI ligands, in agreement with the photocatalytic experiments with MEMI loadings above 75 equiv.

Next, we sought to investigate the influence of the MEMI ligands on the stabilization of the adsorbed *CO_2_^*δ*−^ by analyzing the noncovalent interactions (NCIs) using the Critic2 software.^58,59^ This approach allows for the pseudo-quantitative measurement of intermolecular interactions including electrostatic interactions, H-bonding, van der Waals interactions and steric effects, and has been successfully applied to the coverage analysis of other functionalized QDs.^60^ The NCI isosurfaces responsible for the stabilization of the *CO_2_^*δ*−^ on the ZnSe|MEMI system are shown in Fig. 5B, while the breakdown of these interactions is presented in Fig. S20.† Notably, two distinct interactions stand out as the most attractive ones, corresponding to a π–p interaction between the aromatic imidazole ring and the O_A_ 2p orbital, and a H-bonding interaction between the imidazole ring of a neighboring MEMI and O_B_. These strong attractive interactions are followed by three relatively weaker attractive interactions, associated to longer H-bonding and van der Waals interactions, and three weak repulsive interactions, mainly due to steric effects. Electrostatic interactions induced by the positive charge of the imidazolium moiety were found to have a lower influence. Importantly, the combination of these attractive and repulsive NCIs, which to our knowledge have not been computationally quantified to date, are essential for the stabilization of the *CO_2_^*δ*−^ intermediate, as confirmed by the observed desorption of CO_2_ upon removal of either the surface ligands or the photogenerated electron. Hence, we conclude that both the MEMI ligands and the photogenerated electron work cooperatively to activate and stabilize CO_2_ on the ZnSe-QD surface.

An alternative way to activate CO_2_, commonly proposed in the literature, is *via* a PCET to form *COOH.^54^ A schematic representation of this process, and the subsequent reduction to *CO from both *COOH and *CO_2_^*δ*−^, is presented in Fig. 5C (paths *a* and *b*, respectively). The likelihood of these reaction pathways on the ZnSe|MEMI system was assessed leading to the Gibbs energy profiles shown in Fig. 5D. According to our calculations, the CO_2_ to CO reduction through the two consecutive PCETs (path *a*, Fig. 5D) is rendered very unlikely based on the high energy required for the initial CO_2_ activation, *i.e.* 2.14 eV. In contrast, the activation of CO_2_*via* the photogenerated electron-mediated mechanism (path *b*, Fig. 5D) is predicted to require a considerably lower energy, *i.e.* 0.77 eV, making this pathway feasible under experimental conditions. The feasibility of path *b* proceeding *via* a rate-limiting ET step to form *CO_2_^*δ*−^ is further supported by experiments conducted at a lower pH, which only accelerated the HER and not CO formation (Fig. S22 and Table S5†).

Once *CO_2_^*δ*−^ is formed *via* path *b*, the reaction may proceed through an exergonic PCET which yields a *COOH intermediate with a negative charge density (*COOH^*δ*−^) as revealed by the Bader charge analysis (Fig. S21†). This species, which lies 0.62 eV above the separate reactants, has also been predicted to be a key intermediate in the CO_2_ reduction catalyzed by cobalt complexes.^61^ The generated *COOH^*δ*−^ intermediate subsequently undergoes a second PCET step which requires only 0.03 eV and results in the desorption of both CO and H_2_O, leading to the regeneration of the ZnSe|MEMI with the photogenerated electron. Overall, the highest energy point in path *b* corresponds to the initial activation of CO_2_ to form *CO_2_^*δ*−^, highlighting the importance of the cooperative effect between the MEMI ligands and the photogenerated electron in the stabilization of this intermediate. Future spectroscopic investigations may further elucidate the reaction mechanism.

## Conclusions

We report a simple organic surface modification strategy to enhance the photocatalytic CO_2_ to CO reduction activity of inexpensive and benign ZnSe QDs. Immobilization of an imidazolium moiety promotes CO formation while suppressing the competing HER on the QD surface. We thereby demonstrate that colloidal QDs can be activated for CO_2_ reduction by modifying the chemical secondary environment through design of a dual functional organic capping ligand without the requirement of an additional transition metal co-catalyst. We also show that the CO selectivity can be modulated with the imidazolium loading, yielding up to a 13-fold increase compared to the non-functionalized ZnSe–BF_4_. Finally, we provide mechanistic insights through TA spectroscopy and periodic DFT calculations, which pinpoints the (unpassivated) Zn atoms of the QD surface as the active sites for both the HER and CO_2_ to CO reduction. The imidazolium ligands partially passivate the Zn surface sites and hinder the competing HER while activating the remaining sites for CO_2_ reduction. This process is shown to involve a photo-excited QD which renders a *CO_2_^*δ*−^ species stabilized by the surrounding imidazolium group on the QD surface as the key reaction intermediate. This work not only advances the understanding of interactions of imidazolium groups with CO_2_ reduction intermediates but can also open new routes in the surface design of photocatalysts without the use of precious metals or synthetically demanding molecular co-catalysts.

## Author contributions

C. D. S., L. H., M. G.-M. and E. R. designed the project. C. D. S. prepared and characterized the ZnSe particles and conducted photocatalytic experiments. K. E. D. and C. D. S. prepared and characterized the capping ligands. E. M.-T. performed all the DFT studies. N. E. recorded transient absorption spectra. C. D. S, K. S., Z. H. and O. A. S. developed and carried out NMR and ITC titration experiments. A. W. and C. D. S. developed the continuous-flow methodology and data evaluation. All authors analyzed the data, discussed the results and assisted with the manuscript preparation. L. H., M. G.-M. and E. R. supervised the project.

## Conflicts of interest

The authors declare no conflict of interest.

## Supplementary Material

SC-012-D1SC01310F-s001
