# Supplementary material for: Imidazolium-modification enhances photocatalytic CO2 reduction on ZnSe quantum dots
Source: Chem Sci. 2021 May 17;12(26):9078–87. doi: 10.1039/d1sc01310f (PMC8261709; doi:10.1039/d1sc01310f)
Supplement: SC-012-D1SC01310F-s001 [file SC-012-D1SC01310F-s001.pdf]

Electronic Supplementary Material (ESI) for Chemical Science.
This journal is © The Royal Society of Chemistry 2021

Supporting Information

Imidazolium-modification enhances photocatalytic CO_2 reduction on ZnSe quantum dots

Constantin D. Sahm[1], Eric Mates-Torres[2], Nora Eliasson[3], Kamil Sokołowski[1,4,5], Andreas Wagner[1], Kristian E. Dalle[1], Zehuan Huang[1,4], Oren A. Scherman[1,4], Leif Hammarström[3*], Max García-Melchor[2*] and Erwin Reisner[1*]

[1] Yusuf Hamied Department of Chemistry, University of Cambridge, Lensfield Rd, Cambridge, CB2 1EW, United Kingdom
[2] School of Chemistry, CRANN and AMBER Research Centres, Trinity College Dublin, College Green, Dublin, 2, Ireland
[3] Department of Chemistry– Ångström Laboratory, Uppsala University, Box 523, 751 20, Uppsala, Sweden
[4] Melville Laboratory for Polymer Synthesis, Department of Chemistry, University of Cambridge, Lensfield Rd, Cambridge, CB2 1EW, United Kingdom
[5] Institute of Physical Chemistry, Polish Academy of Sciences, Kasprzaka 44/52, 01-224 Warsaw, Poland

Materials. Zinc stearate (purum, Sigma-Aldrich), octadecene (90% techn., Sigma-Aldrich), trioctylphosphine (90%, Sigma-Aldrich), selenium powder (99%, Sigma-Aldrich), *n*-heptane (99%, Aldrich), methanol (99.8%, Fisher-scientific), acetone (laboratory reagent grade, Fisher-scientific), 1-butanol (99%, Alfa Aesar), trimethyloxonium tetrafluoroborate (96%, Sigma-Aldrich), L-ascorbic acid (99%, Sigma-Aldrich), 1-ethyl-3-methylimidazolium tetrafluoroborate (99%, Sigma-Aldrich), 1-Butanethiol (99%, Sigma-Aldrich) were used as received. Anhydrous solvents were purchased from Acros Organics with the following purities: $CHCl_3$ (99.9%), *N*,*N*-dimethylformamide (DMF, 99.8%). All aqueous experimental solutions were prepared with ultrapure water (DI water; Milli-Q®, 18.2 MΩ cm). $^{13}CO_2$ (>99 atom% ^{13}C) was purchased from Sigma Aldrich.

Powder X-ray diffraction (XRD). XRD was conducted using an X'Pert PRO by PANalytical BV instrument using CuK_α irradiation.

Infrared spectroscopy. Gas-phase transmission IR spectra of the photoreactor headspace were recorded on a Thermo Scientific Nicolet iS50 FT-IR spectrometer in transmission mode. ATR-IR spectra were recorded on the same spectrometer in ATR mode.

Transmission electron microscopy (TEM). TEM images were collected using a Thermo Scientific (FEI) Talos F200X G2 TEM, operating at an accelerating voltage of 200 kV. Samples were prepared by drop-casting a dilute QD-solution on holey-carbon coated Cu grids followed by evaporation of the solvent.

UV−Vis spectroscopy. UV−Vis spectra were recorded on an Agilent Cary 60 UV−Vis spectrophotometer using quartz glass cuvettes (1 cm path length).

^{1}H Nuclear magnetic resonance (NMR) titration experiments. ^{1}H-NMR spectra were recorded on a Bruker AVANCE 500 with a TCI Cryoprobe system (500 MHz). Chemical shifts are recorded in D_2O in ppm with the internal reference set to the solvent peak at $\delta = 4.80$ ppm. In a typical NMR titration experiment, 1 mL of a 5 µM ZnSe-QD solution in D_2O is mixed with specific amounts of capping ligand stock solution (typically 10 µL of a 0.5 mM in D_2O) inside an NMR tube under an atmosphere of N_2. All NMR titration spectra were measured with 256 scans.

Calculation of the surface coverage. The surface coverage was calculated according to the following formula:

$$Surface\ coverage = \frac{N_{MEMI} \cdot SA_{MEMI}}{SA_{QD}} = \frac{N_{MEMI} \cdot SA_{MEMI}}{4\pi r^2}$$

The surface area of a QD (SA_{QD}) was approximated using the equation for the surface area of a sphere taking into account the average QD diameter of ~4.5 nm. For a MEMI ligand, a surface occupation area (SA_{MEMI}) of ~1 nm was assumed and then the number of MEMI ligands (N_{MEMI}) and their surface occupation was divided by the surface area of a QD.

Isothermal titration calorimetry (ITC). ITC experiments were carried out using a Malvern Microcal Auto-ITC200. During a typical ITC titration, ZnSe-BF_4 QD (1 or 2 µM, 200 µL) solution was placed inside the sample cell and the capping ligand solution (0.5 or 1 mM) in the syringe is stepwise titrated to the QD solution. In order to avoid background signals from DMF (as native solvent of the QDs), the concentration of DMF was kept constant (3.12 %, v/v) in the solutions within the cell and the syringe. Each experiment was conducted at 298.15 K and allowed to equilibrate prior to an initial 60 s delay. One titration experiment consisted of 1 injection of 0.6 µL and 32 consecutive injections of 1.2 µL with 90 s intervals between injections. Reference titrations were conducted to determine any significant heat of dilution between the solvent mixtures, ligand solution that may have accounted for signal in the final ligand QD titrations. Ligand-solvent reference titrations were subtracted from ligand QD titrations. The obtained ITC curves were fitted by MicroCal Analysis Centre software using one set of sites binding model. The thermograms were fit with the simple independent identical sites model.[1]

Zeta potential. Zeta potential measurements of ZnSe-BF_4 and in the presence of MEMI were conducted using a Malvern Zetasizer Nano ZS90 instrument at 25°C.

External quantum efficiency (EQE). Photocatalysis samples were prepared as stated in the Photocatalysis Section, but under accumulation of products in the headspace using a flat-sided quartz cuvette (1 cm path length, airtight) as the photoreactor. The sample was purged with CO_2 (containing 2% CH_4 as internal standard) and primed by irradiation for 25 min with a solar light simulator as stated above. The cuvette was then purged again with CO_2/CH_4 (2 %) and irradiated with monochromatic light (λ = 400±5 nm, A = 0.80 cm^2) using an LOT Quantum Design MSH-300 monochromator. Aliquots of headspace gas were taken periodically and analysed by gas chromatography. The EQE was calculated according to:

$$\text{EQE (\%)} = \frac{2n \times N_A \times h \times c}{t_{\text{irr}} \times \lambda \times I \times A} \times 100$$

Where n is the amount of produced CO or H_2 per time, N_A is Avogadro's constant, h is the Planck constant, c is the speed of light, t_{irr} is the irradiation time, λ is the irradiation wavelength, I is the irradiation intensity and A is the irradiated area.

Preparation of 3-(2-mercaptoethyl)-1-methyl-imidazolium bromide (MEMI).

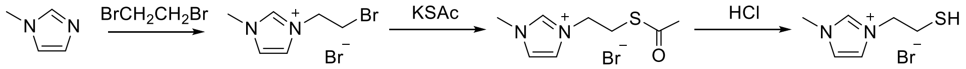

3-(2-Bromoethyl)-1-methylimidazolium bromide was synthesized according to an adapted literature procedure.[2] 1-Methylimidazole (4.0 mL, 50 mmol) was dissolved in a solution of anhydrous diethyl ether (25 mL) and 1,2-dibromoethane (20 mL, 230 mmol) slowly added. The resulting solution was stirred slowly at room temperature (RT) under an inert atmosphere and a colorless crystalline product deposited over several days. The solution was removed using a canula filter, and the solid 3-(2-bromoethyl)-1-methylimidazolium bromide washed with dry diethyl ether. Yield: 8.65 g, 64%. ^{1}H NMR (400 MHz, DMSO-d^6) δ (ppm) = 9.29 (s, 1H), 7.87 (m, 1H), 7.78 (m, 1H), 4.64 (t, *J* = 5.9 Hz, 2H), 3.96 (t, *J* = 5.9 Hz, 2H), 3.90 (s, 3H). ^{13}C NMR (101 MHz, DMSO-d^6) δ (ppm) = 136.99, 123.71, 122.34, 50.04, 35.89, 31.56.

3-(2-(Acetylthio)ethyl)-1-methyl-imidazolium bromide was prepared using a modified literature procedure.[3] Solid potassium thioacetate (846 mg, 8.6 mmol) was added to 3-(2-bromoethyl)-1-methyl-imidazolium bromide (2.0 g, 7.4 mmol) in anhydrous acetonitrile (20 mL) and the resulting mixture stirred overnight under reflux. After cooling to RT the solution was removed from precipitated KBr by canula filtration, the solvent removed in vacuo, and the residue thoroughly dried to afford pale-yellow-white 3-(2-(acetylthio)ethyl)-1-methyl-imidazolium bromide. Yield: 1.89 g, 96%. ^{1}H NMR (400 MHz, DMSO-d^6) δ (ppm) = 9.19 (s, 1H), 7.79 (m, 1H), 7.71 (m, 1H), 4.36 (t, *J* = 6.4 Hz, 2H), 3.86 (s, 3H), 3.32 (t, *J* = 6.4 Hz, 2H), 2.35 (s, 3H). ^{13}C NMR (101 MHz, DMSO-d^6) δ (ppm) = 194.51, 136.93, 123.55, 122.56, 48.06, 35.80, 30.55, 28.59. ATR-IR (neat): 3403*br*, 3140*m*, 3060*s* (C-H), 2981*s*, 2854*w* (CH_2), 1687*s* (C=O), 1577*s* (Im^+), 1560*s* (Im^+), 1454*m*, 1426*m*, 1356*m*, 1337*w*, 1300*m*, 1277*w*, 1220*w*, 1166*s*, 1130*m*, 1107*m*, 1044*w*, 1021*w*, 1008*w*, 952*br*, 757*br*, 719*w*, 700*w*, 680*w*, 646*m*, 620*m*.

3-(2-(Acetylthio)ethyl)-1-methyl-imidazolium bromide was added into a 50 mL Schlenk flask and dried in vacuo. Hydrochloric acid (HCl, 1M, 17.3 mL) was degassed by purging with nitrogen for 1 h and added to the flask. The resulting mixture was stirred for 63 h under inert gas atmosphere at 40 °C. The reaction product was isolated by evaporating the by-products and solvents. The 3-(2-Mercaptoethyl)-1-methyl-imidazolium bromide produced was kept under inert conditions before use in order to prevent aerobic disulfide formation. Yield was not obtained accurately due to the viscous, hygroscopic nature of the compound. ^{1}H NMR (400 MHz, DMSO-d^6) δ 9.16 (s, 1H), 7.78 (m, 1H), 7.73 (m, 1H), 4.33 (t, *J* = 6.5 Hz, 2H), 3.87 (s, 3H), 2.95 (dt, *J* = 8.4 Hz, 6.5 Hz, 2H), 2.68 (t, *J* = 8.4 Hz, 1H). ^{13}C NMR (100 MHz, DMSO-d^6) δ (ppm) = 136.79, 123.57, 122.34, 51.25, 35.78, 23.91. ATR-IR (neat): 3403*br*, 3138*m*, 3072*s* (C-H), 2851*w* (CH_2), 2425*br*, 1975*w*, 1630*m* (C=C), 1577*s* (Im^+), 1560*s* (Im^+), 1451*m*, 1425*m*, 1384*w*, 1359*w*, 1336*m*, 1300*m*, 1281*w*, 1251*w*, 1164*s*, 1089*w*, 1021*w*, 960*br*, 827*br*, 751*br*, 711*w*, 666*w*, 643*m*, 620*m*. Elemental analysis calcd. (%) for $C_6H_{11}N_2S_1Cl_{0.5}Br_{0.5}$ x $(H_2O)_{1.25}$: C 32.26, H 6.09, N 12.54, found C 32.15, H 6.17, N 12.33. HRMS: (m/z) calcd. for $C_6H_{11}N_2S^+$: 143.0637 $[M]^+$; found 143.0627.

Preparation of 3-(2-mercaptoethyl)-1,2-dimethyl-imidazolium bromide (M-MEMI).

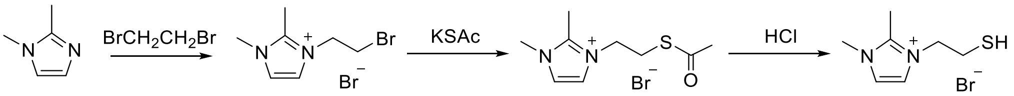

3-(2-Bromoethyl)-1,2-dimethyl-imidazolium bromide was synthesized according to literature procedure[4] and re-crystallized in a methanol/ethyl acetate mixture (50:50). Yield (after recrystallisation): 2.77 g (65%). ^{1}H NMR (400 MHz, DMSO-d^6) δ (ppm) = 7.72 (d, J = 2.1 Hz, 1H), 7.68 (d, J = 2.1 Hz, 1H), 4.60 (t, J = 6.0 Hz, 2H), 3.90 (t, J = 6.0 Hz, 2H), 3.79 (s, 3H), 2.63 (s, 3H). ^{13}C NMR (101 MHz, DMSO-d^6) δ (ppm) = 144.97, 122.44, 121.14, 48.54, 34.91, 31.29, 9.53.

3-(2-(Acetylthio)ethyl)-1,2-dimethyl-imidazolium bromide was synthesized according to modified literature procedure.[5] 3-(2-bromoethyl)-1,2-dimethyl-imidazolium bromide (0.5 g) was combined with potassium thioacetate (201.1 mg) in anhydrous acetonitrile (10 mL) and the resulting mixture was stirred overnight under reflux. KBr was removed by filtration and the off-white precipitate was collected by drying in vacuo. Yield: 330 mg (94%). ^{1}H NMR (400 MHz, DMSO-d^6) δ (ppm) = 7.63 (d, J = 2.0 Hz, 1H), 7.61 (d, J = 2.0 Hz, 1H), 4.30 (t, J = 6.7 Hz, 1H), 3.75 (s, 3H), 3.24 (t, J = 6.7 Hz, 1H), 2.62 (s, 3H), 2.34 (s, 3H). ^{13}C NMR (100 MHz, DMSO-d^6) δ (ppm) = 194.81, 144.83, 122.16, 121.32, 46.71, 34.74, 30.48, 27.96, 9.35. ATR-IR (neat): 3414*br*, 3400*w*, 3174*w*, 3111*m*, 3073*s* (C-H), 3042*m*, 2987*w*, 2966*s*, 2954*m*, 2917*w*, 2906*w*, 2891*m*, 2760*w*, 2430*w*, 1740*w*, 1705*s* (C=O), 1670*w*, 1587*m*, 1533*s*, 1514*m*, 1470*m*, 1458*w*, 1432*m*, 1415*s*, 1393*w*, 1372*w*, 1360*m*, 1349*s*, 1340*m*, 1310*w*, 1268*s*, 1245*w*, 1167*m*, 1119*s*, 1094*s*, 1062*w*, 1045*w*, 1028*m*. 999*w*, 979*w*, 967*s*, 954*m*, 876*w*, 779*s*, 732*m*, 706*w*, 680*m*, 667*s*, 614*s*, 585*w*, 529*w*, 495*w*, 480*w*, 445*w*.

3-(2-mercaptoethyl)-1,2-dimethyl-imidazolium bromide (M-MEMI) was synthesized by loading 3-(2-(acetylthio)ethyl)-1,2-dimethyl-imidazolium bromide (120 mg) into a Schlenk flask and drying in vacuo. Hydrochloric acid (HCl, 1 M, 10 mL) was degassed by purging with nitrogen for 1 h and added to the flask. The resulting mixture was stirred for 98 h under inert gas atmosphere at 45 °C. The reaction product was isolated by evaporating the by-products and solvents and was kept under strict inert conditions before use in order to prevent aerobic disulfide formation. ^{1}H NMR (400 MHz, DMSO-d^{6}) δ (ppm) = 7.65 (d, J = 2.1 Hz, 1H), 7.63 (d, J = 2.1 Hz, 1H), 4.28 (t, J = 6.7 Hz, 2H), 3.76 (s, 3H), 2.89 (m, 2H), 2.69 (m, 1H), 2.61 (s, 3H). ^{13}C NMR (100 MHz, DMSO-d^{6}) δ (ppm) = 144.72, 122.29, 121.06, 49.83, 34.71, 23.65, 9.44. ATR-IR (neat): 3414*br*, 3174*w*, 3109*m*, 3058*s* (C-H), 3020*m*, 2987*w*, 2957*s*, 2945*w*, 2922*m*, 2441*m*, 1773*w*, 1670*w*, 1588*m*, 1537*s*, 1514*m*, 1456*s*, 1432*w*, 1413*s*, 1373*m*, 1362*m*, 1344*m*, 1305*w*, 1266*m*, 1241*m*, 1222*w*, 1165*m*, 1119*m*, 1094*w*, 1058*w*, 1034*m*, 951*w*, 889*w*, 814*m*, 789*s*, 731*m*, 703*w*, 672*w*, 658*m*, 626*w*, 579*w*, 478*w*, 444*w*. Elemental analysis calcd. (%) for $C_7H_{13}N_2S_1Br$ x $(H_2O)_{0.06}$: C 35.29, H 5.55, N 11.76, found C 35.57, H 5.57, N 11.49. HRMS: (m/z) calcd. for $C_7H_{13}N_2S^+$: 157.0794 [M]$^+$; found 157.0788.

ZnSe-St. Stearate capped ZnSe-QDs were prepared by a modified literature procedure[6,7] as follows: Zinc stearate (758 mg), Se powder (79 mg) and octadecene (65 mL) were added to a 250 mL three-necked flask and degassed for 2 h at 50 °C under vacuum. The reaction was triggered by raising the temperature to 300 °C under a N_2 atmosphere, resulting in an initially colorless reaction mixture that turned progressively yellow. To monitor particle growth, aliquots (100 µL) were taken periodically, diluted with $CHCl_3$ to 1 mL total volume, filtered with a syringe filter (Merck Millex-GN, 0.20 µm nylon membrane) and analysed by UV-vis spectroscopy. After 2 h 10 min (counted from the time when the temperature was raised above 50 °C) the reaction was stopped by removing the heating and blowing N_2 into the flask. The particles were precipitated using an acetone/methanol mixture (20:80), followed by centrifugation (7,000 rpm, 10 min). The residue was washed with n-butanol and twice with methanol and re-dispersed in $CHCl_3$.

ZnSe-BF$_4$. Ligand-free ZnSe QDs were prepared by reactive ligand removal using a modified literature procedure[7] for CdS stripping. A ZnSe-St solution (3 mL, in $CHCl_3$) was dried *in vacuo*. Under a N_2 atmosphere, the residue was re-dispersed in a mixture of anhydrous $CHCl_3$ (3 mL) and anhydrous DMF (0.2 mL). Aliquots of stripping agent (Me_3OBF_4, 1.0 M in acetonitrile, typically 3.5 mL) were added slowly until the particles precipitated. The resulting ligand-free particles were centrifuged (7,000 rpm, 15 min), dried in air for 1 min, and re-dispersed in DMF (3-4 mL). The resulting slightly cloudy solution of ZnSe-BF$_4$ in DMF was further purified by centrifugation (7,000 rpm, 15 min) to give a black precipitate, a clear yellow solution and a cloudy white phase on top. The black precipitate and white phase were removed and the clear yellow solution was used for characterisation and photocatalytic experiments. Thus-prepared ZnSe-BF$_4$ can be handled in air for hours without decomposition but will gradually degrade over several days. To prevent degradation, the ZnSe-BF$_4$ solution was degassed by 4 freeze-pump-thaw cycles and stored under N_2 in the dark at 4 °C. The mean particle size was determined from TEM images. To calculate the QD concentration in the stock solution, the Zn^{2+} and Se^{2-} concentration determined by ICP-OES, was divided by the number of Zn atoms per QD based on the mean particle diameter (d = 4.54 nm) and the bulk density of ZnSe (5.262 g cm^{-3}).

Photocatalytic CO_2 reduction

Sample preparation. A ZnSe-BF$_4$ stock solution (64.1 µM in DMF, 23.40 µL) and a capping ligand solution (5.0 mM in water, typically 30 µL) were added to a Pyrex glass photoreactor (Chromacol 10-SV, Fisher Scientific) containing a magnetic stirrer bar. The mixture was diluted with ascorbic acid (AA, 0.1 M in water, pH adjusted to 7 with NaOH) to a total solution volume of 3 mL. $NaHCO_3$ powder (25 mg) was further added to increase the pH to 8.3. A screening for sacrificial electron donors (ascorbic acid, citric acid, EDTA, Na_2S, Na_2SO_3, TEOA, methanol) only yielded good photocatalytic activity for AA, hence the latter was used in the current study. The photoreactor was then sealed with a rubber septum and pierced with two needles (inlet and outlet). This catalyst assembly method leads to the presence of some solvent residues in the mixture used for photocatalysis (0.8% v/v DMF), and other solvent residues (<0.07% v/v for MeOH, BuOH, acetonitrile, see Figure S2) which were found to not affect the photocatalytic activity significantly.

Constant flow-setup with automated product quantification. The inlet of the photoreactor was connected to a Mass Flow Controller (Brooks GF040) supplying a stream of CO_2 (CP Grade, BOC, humidified with a water bubbler) with a flow rate of 4.0 sccm. The flow rate at the GC outlet was verified prior to the experiment with an Alicat gas flow meter to avoid gas leakage. The outlet of the photoreactor was connected to a flow selection valve controlled by a Shimadzu Tracera GC-2010 Plus gas chromatograph for product quantification of the gaseous reaction products (see below). Six samples (two triplicates of identical conditions) were typically analyzed in parallel. Upon purging with a constant stream of CO_2, the solution pH decreased to 6.5 due to saturation with CO_2. The photoreactor was purged for a further 45 min in the dark and sampled via online GC quantification. The first two injections of each sample were used to determine a “background” peak which was subtracted from further injections. The photoreactor was then placed in a water bath maintained at 25 °C, stirred and irradiated by a solar light simulator (Newport Oriel, AM 1.5G, 100 mW cm^{-2}). The six samples were evenly distributed within the light simulator to account for possible variations of the light intensity depending on the position in the simulator. UV irradiation was filtered with a 400 nm cut-off filter (UQG).

For ^{13}C isotopic labelling, photocatalysis experiments were performed as described above, but with accumulating products in the headspace under steady-state conditions and using $^{13}CO_2$ as the headspace gas. After 1000 min (16.7 h), the photoreactor headspace was transferred to an evacuated gas IR cell (SpecAc, 10 cm path length, equipped with KBr windows) and a high-resolution gas-phase transmission spectrum was collected.

The Shimadzu Tracera GC-2010 Plus gas chromatograph (GC) used a barrier discharge ionization detector, kept at 300 °C, and was equipped with a Hayesep D (2 m * 1/8'' OD * 2 mm ID, 80/100 mesh, Analytical Columns) pre-column and a RT-Molsieve 5A (30 m * 0.53 mm ID, Restek) main column in order to separate H_2, O_2, N_2, CH_4 and CO while hindering CO_2 and H_2O to reach the Molsieve column. He carrier gas (Grade 5.0, BOC) was purified (HP2-220, VICI) prior to entering the GC. The column temperature was kept at 85°C. The gaseous flow from the flow selection valve was passed through a loop (volume 1.0 mL) and injected approximately every 4.25 min into the GC. Effectively, each individual sample was injected every 25.5 min. The GC calibration was performed with a known standard for H_2, CO and CH_4 (2040 ppm H_2/2050 ppm CO/2050 ppm CH_4 in balance gas CO_2, BOC) by diluting the mixture with pure CO_2.

Data analytics. The data was processed and visualized using the statistical programming language *R* with the *tidyverse* library.[9,10] First, the flow rates were corrected by subtracting a "background" peak obtained in the dark prior to irradiation (we noticed a marginal CO background peak depending on the residual amount of oxygen present in the sample stream – a feature of the gas chromatograph and not the sample). Second, the momentary product evolution rate corresponding to each injection was calculated using the following formula. ˙

$$product\ evolution\ rate = \frac{p * \dot{V} * \frac{Area\ GC}{f_i}}{R\ T}$$

where p is the pressure in the photoreactor (ambient pressure, 101325 Pa), $\dot{V}$ is the flow rate (4.0 sccm), R is the universal gas constant, T is the temperature (298 K) and f_i is the response factor for each gas determined by the calibration procedure. Third, the total amount of evolved product was calculated using trapezoidal integration of the product evolution rates. The three independent replicates of identical conditions were averaged by calculating the mean and standard deviation over irradiation time and sample. For visual display, the actual values for each sample are plotted as transparent scatter, whereas the mean is represented as a smoothened continuous line. In addition, the standard deviation is visualized by the shaded area surrounding the mean where the transparency is proportional to the standard deviation. Specifically, the calculated standard deviation is used to compute a Gaussian density for that standard deviation, plotting a cloud with the opacity proportional to the density. This appears as a vertical "cloud" of uncertainty[11]. The maximum of the uncertainty cloud is set to 1.5 standard deviations.

Transient absorption (TA) spectroscopy

Femtosecond Transient Absorption.

Femtosecond transient absorption (fs-TA) experiments were performed using a Ti:sapphire (Ti:Sa) based amplifier (Libra, Coherent Inc.) with an integrated Ti:Sa Kerr-lens mode-locked fs-seed laser and Q-switched Nd:YLF optical pump. The system operates at 800 nm (1.5 mJ, FWHM: 45 fs) with a 3 kHz repetition rate. The laser fundamental was split into a high-intensity pump and probe by a beam splitter. The probe was directed towards the UV-Vis-NIR sample chamber (TAS, Newport Corp., Ultrafast Systems) where the super-continuum (UV-Vis/NIR) was generated from a calcium fluoride/sapphire (CaF_2/Al_2O_3) crystal. The pump was frequency-doubled (~400 nm) using a birefringent barium borate (BBO) crystal and attenuated (25-400 µW, 20-300 nJ/pulse) in the sample chamber using a neutral density filter. Prior to the sample cell, the pump was interrupted periodically by a chopper so that every second pulse were allowed to generate an excited population. The pump-probe delay time ($\Delta_{p\text{-}p}t \leq 8$ ns) was controlled by a mechanical optical delay line in the probe beam path, allowing the time-evolution of the differential absorbance between the pump induced excited state spectrum and the unpumped ground state spectrum to be recorded (silicon diode array: home built, Newport custom made). The instrument response function (IRF) was typically 140-160 fs and the number of scans for each measurement was limited to <7 scans (1500 integration) to avoid photodamage.

Sample preparation and Measurements

The ZnSe-BF_4 (batch B1/B2: 77.3 µM/754 µM in DMF) quantum dots (QDs) and MEMI capping ligand (50 mM/100 mM in water) stock solutions were stored in Schlenk flasks under an inert atmosphere (~4°C). The samples (ZnSe; ZnSe | MEMI, 1:100; ZnSe | AA, 1:1000; ZnSe | MEMI | AA, 1:100:1000) were prepared with a final ZnSe concentration of 5-10 µM (≥ 87 % H_2O vs DMF) and put in quartz cuvettes (1 mm pathlength) sealed with teflon caps and parafilm. All samples were prepared fresh on the day of measurements, including the AA stock solution (AA/NaOH, 50 mM, pH: ~6.7, purged with

inert gas). It should be noted that the QDs are easily prone to agglomeration, but all measurements were repeated on multiple occasions on both of the individually prepared QD batches (B1 and B2). All features and dynamics reported in the main article (pump energy: 70 nJ: linear regime, see Figure S15) were consistent across all measured samples of the same type, apart from in the neat ZnSe QD, where slight deviations were observed in the wavelength shift magnitude of the bleach maximum (Figure 4D vs Figure S16D). With MEMI present, the $\Delta\lambda_{bleach}$ was consistent across all measurements. Slight sample-to-sample variations are not surprising considering the differences between the spectral response reported for these QDs in Reisner (2018) and the present study (see main article), likely owing to the TA signals′ sensitivity to the surface conditions (see main article) of the ligand stripped QDs. The shape of the superimposed transients can therefore be expected to vary, reflecting differences in the relative weights of the signal amplitudes.

Data Analysis

All data treatment was performed in SurfaceXplorer Data Analysis Software (Ultrafast systems). Initially, the individual scans were analyzed carefully to check for inconsistencies and the build up of photoproducts. For all TA reported spectra, the background and scattered pump light was extracted from the compiled datasets (scan average). The spectra were subsequently fitted with a polynomial function for chirp-correction, which ensures that time-zero is set equal at all probe wavelengths.

Computational methods

DFT calculations reported in this study were performed using the Perdew−Burke−Ernzerhof (PBE) functional[12] as implemented in the Vienna *Ab Initio* Simulation Package (VASP) software, version 5.4.4.[13] The core electrons of Zn, Se, S, O, N, C and H atoms were replaced by projector-augmented wave (PAW) pseudopotentials,[14] while their valence electrons were expanded in plane waves with a kinetic energy cut-off of 500 eV, using a scaling constant of 0.05 Å and a force-based convergence criteria of 0.015 eV $Å^{-1}$. Dispersion corrections were added using the zero-damping DFT-D3 method by Grimme[15] to account for non-covalent interactions (NCIs). The bulk structure of ZnSe was retrieved from the Materials Project database,[16] and the equilibrium lattice constant was optimized by fitting the energy of a number of bulk ZnSe structures with lattice parameters ranging between ±5% of its initial value to the Birch-Murnaghan equation of state, sampling the reciprocal space using Γ-centred k-point grids of 3×3×3, 5×5×5, 7×7×7 and 9×9×9. Ultimately, a Γ-centred k-point grid of 5×5×5 was selected with a k-point density of 0.87 Å for surface calculations following a convergence criterion of 1 meV $atom^{-1}$. Molecules were calculated at Γ-point with at least 15 Å of vacuum along the three axes.

The energies of the 4-layered slabs of the (111), (200), (220) and (311) facets were calculated with a vacuum of at least 15 Å perpendicular to the surface. The bottom two layers were fixed to their bulk positions, whereas the two topmost layers were allowed to relax. This configuration was chosen based on walltime and energy convergence criteria. Surface energies (γ_i) were calculated with the following formula:

$$\gamma_i = \frac{E_{slab} - n \times E_{bulk}}{2A}$$

Where E_{slab} corresponds to the calculated energy of the optimized 4-layer slab, $n \times E_{bulk}$ is the energy of the optimized bulk multiplied by the number of bulk units in the slab, and A is the sectional surface area. The calculated γ_i values for the different facets are presented in Table S6. The Wulff construction method was applied to determine the ZnSe QD morphology in equilibrium conditions, using the Wulff module implemented in the Pymatgen library.[17] After confirming that MEMI binds only on the coordinatively unsaturated Zn surface atoms of ZnSe through the thiol group, we proceeded to assess the relative stability of different ligand concentrations by adsorbing 1 or 2 MEMI ligands on the surface Zn atoms of ZnSe(220) surfaces with the following multiplicities: $p(1\times1)$, $p(2\times1)$ and $p(2\times2)$, effectively achieving coverages of 12.5 %, 25 %, 50 % and 100 %, respectively (representing the percentage of surface Zn sites saturated with MEMI ligands). The adsorption energy of the MEMI ligands on these surfaces was calculated as follows:

$$\Delta E = E_{ZnSe*n\times MEMI} - E_{ZnSe} - n \times E_{MEMI}$$

Where $E_{ZnSe*n\times MEMI}$ corresponds to the energy of the MEMI-covered ZnSe(220) slab, E_{ZnSe} is the energy of the bare ZnSe slab and $n \times E_{MEMI}$ is the energy of the isolated MEMI ligand multiplied by the number of ligands in the unit cell. The normalized energies of adsorption of each coverage are depicted in Figure S18. The most stable coverage of 50 % (ZnSe | MEMI) was used for mechanistic studies, represented by two MEMI ligands, each adsorbed on one of the four Zn surface sites in a $p(2\times1)$ ZnSe(220) surface; further adsorption of MEMI on either of the two additional sites was found to be endergonic due to the steric hindrance imposed by the imidazolium rings of the adsorbed ligands. A similar method was employed to calculate the adsorption of H atoms on the surface, wherein the energy of the hydrogen was replaced by one-half of the energy of molecular hydrogen following the computational hydrogen electrode model.[18] Similarly to the MEMI ligands, our simulations indicate that H atoms adsorb predominantly atop the surface Zn atoms. Furthermore, calculations show that the presence of a photogenerated electron is essential to lower the H adsorption energy with respect to the neutral system, from 2.15 eV to 0.36 eV on the bare surface (Figure S19, A) and from 1.87 eV to 0.15 eV on the ZnSe | MEMI system (Figure S19, B), which is theorized to favor HER.[19] Hence, the presence of a photogenerated electron is required to

promote HER on the bare and ZnSe | MEMI systems, in agreement with experiments, and hindering of HER on the ZnSe | MEMI system stems from the blockage of Zn active sites due to the stronger adsorption energy of MEMI compared to H.

Gibbs corrections to the energy were computed including the zero-point energy (ZPE), vibrational enthalpy and entropy terms obtained by means of the Thermochemistry module implemented in the Atomic Simulation Environment (ASE) package, at the experimental temperature of 298 K. In the calculation of the Gibbs energy corrections for the different molecules, we considered a pH of 6.5, partial pressures of CO_2 of 1 atm and H_2O of 0.035 bar (which is the pressure at which the gas and liquid phases of H_2O are in equilibrium at 300 K), and the experimentally detected concentration of CO of 3μM.

Adsorption Gibbs energies of the different reaction intermediates on the ZnSe | MEMI system were calculated as follows:

$$\Delta G_{*CO_2^{\delta-}} = G_{*CO_2^{\delta-}} - G_{*e^-} - G_{CO_2}$$

$$\Delta G_{*COOH^{\delta-}} = G_{*COOH^{\delta-}} - G_{*e^-} - \left(G_{CO_2} + \frac{1}{2}G_{H_2}\right)$$

$$\Delta G_{*COOH} = G_{*COOH} - G_{*} - \left(G_{CO_2} + \frac{1}{2}G_{H_2}\right)$$

$$\Delta G_{*CO} = G_{*CO} - G_{*} - \left(G_{CO_2} + G_{H_2} - G_{H_2O}\right)$$

Where $G_{*CO_2^{\delta-}}$, $G_{*COOH^{\delta-}}$, G_{*COOH} and G_{*CO} are the Gibbs energies of the $*CO_2^{\delta-}$, $*COOH^{\delta-}$, *COOH and *CO intermediates in the ZnSe | MEMI system, respectively. The *COOH and *CO intermediates take part in the pathway in which CO_2 is adsorbed following a PCET step (Figure 5, Path *a* in the main text), while the $*CO_2^{\delta-}$ and $*COOH^{\delta-}$ intermediates belong to the mechanism where the first step involves an electron transfer to activate CO_2 (Figure 5, Path *b* in the main text). G_{*e^-} and G_{*} are the Gibbs energies of the ZnSe | MEMI including or omitting an additional electron for the aforementioned first and second mechanisms, respectively; and G_{CO_2}, G_{H_2} and G_{H_2O} are the Gibbs energies of the CO_2, H_2 and H_2O molecules in the gas phase. Note that a * denotes an adsorbed species. The Gibbs energy of the overall reaction was calculated as follows:

$$\Delta G_R = G_{CO} + G_{H_2O} - \left(G_{CO_2} + G_{H_2}\right)$$

The analysis of the NCIs stabilising the $*CO_2^{\delta-}$ intermediate and facilitating CO_2 activation in the first mechanism were assessed by computing the reduced density gradient, $s(r)$, as a function of the electron density, $\rho(r)$, by means of the Critic2 software,[20] as described elsewhere.[21]

All the computational data reported in this work, including the cartesian coordinates and energies of all the modelled structures, is accessible via the following ioChem-BD online dataset, DOI: 10.19061/iochem-bd-6-36

Supporting Tables

Table S1. Optimization of photocatalytic CO_2 reduction using ZnSe-BF_4 | MEMI. Unless otherwise stated, standard conditions were: 0.5 μM QD, 0.1 M AA/$NaHCO_3$, pH 6.5, 3 mL water under CO_2 flow (4 sccm); 100 mW cm^{-2}, AM 1.5G, λ >400 nm, 10 h irradiation, 25 °C.

Co-catalyst	Ligand loading / μM	$n(H_2) \pm \sigma$ / mmol g_{ZnSe}^{-1}	$n(CO) \pm \sigma$ / mmol g_{ZnSe}^{-1}	CO selectivity[a]
varying the catalyst				
ZnSe	0	71.8 ± 19.7	0.64 ± 0.11	0.9% ± 0.3%
ZnSe | EMIM-BF_4	50	40.2 ± 6.06	1.06 ± 0.06	2.6% ± 0.5%
ZnSe | MEMI	50	29.9 ± 8.52	1.78 ± 0.23	5.8% ± 1.1%
ZnSe | M-MEMI	50	17.3 ± 0.87	2.38 ± 0.19	12% ± 1.1%
ZnSe | BuSH	50	72.1 ± 27.6	0.69 ± 0.19	1.1% ± 0.5%
varying ligand loading				
ZnSe | MEMI	0	71.8 ± 19.7	0.64 ± 0.11	0.9% ± 0.3%
ZnSe | MEMI	12.5	46.9 ± 8.15	1.18 ± 0.22	2.5% ± 0.7%
ZnSe | MEMI	25	38.6 ± 13.0	1.41 ± 0.10	3.8% ± 1.1%
ZnSe | MEMI	37.5	26.0 ± 1.69	1.52 ± 0.14	5.5% ± 0.3%
ZnSe | MEMI	50	29.9 ± 8.52	1.78 ± 0.23	5.8% ± 1.1%

Table S2. Control experiments for the photocatalytic CO_2 reduction using ZnSe | MEMI. Unless otherwise stated, conditions were: 0.5 μM QD, 0.1 M AA/$NaHCO_3$, pH 6.5, 3 mL water under CO_2 flow (4 sccm); 100 mW cm^{-2}, AM 1.5G, λ >400 nm, 25 °C.

description	time / h	$n(CO) \pm \sigma$ / mmol g_{ZnSe}^{-1}	$n(H_2) \pm \sigma$ / mmol g_{ZnSe}^{-1}
standard experiment (ZnSe | MEMI)	10	1.78 ± 0.23	29.9 ± 8.52
no MEMI	10	0.64 ± 0.11	71.8 ± 19.7
no AA	16	not detected	0.05 ± 0.03
no ZnSe QDs	16	not detected	not detected
no light	10	not detected	not detected

Table S3. Formate production. Irradiation time 4 h; The quantification of formate is complicated due to a background formation of formate, presumably from decomposition of ascorbate. In order to differentiate between formate from reduced CO_2 or decomposed ascorbate, the experiment was conducted under an atmosphere of $^{13}CO_2$ and ^{13}formate was quantified via solution ^{1}H-NMR spectroscopy and ^{13}CO via gas chromatography. Conditions were: 0.5 μM QD, 0.1 M AA/$NaHCO_3$, pH 6.5, 2 mL water under CO_2; 100 mW cm^{-2}, AM 1.5G, λ >400 nm, 25 °C

description	$n(CO) \pm \sigma$ / μmol	n(formate) / μmol	Ratio of formate vs CO
ZnSe | MEMI	0.290 ± 0.028	0.009	2.9%

Table S4. External quantum efficiency (EQE) determination for the photocatalytic CO_2 reduction using Zn-Se-BF_4 | M-MEMI (1.0 μM ZnSe-BF_4, 100 μM M-MEMI, in 1.2 mL 0.1 M aq. AA, pH 6.5 under CO_2; A = 0.80 cm^2, λ = 400±5 nm, rt).

time / h	Intensity / mW cm^{-2}	n(CO) / nmol[a]	EQE_{CO} / %	n(H_2) / nmol[a]	EQE_{H2} / %
2	1.0	27±17[b]	0.28±0.18	324±222[b]	3.36±2.30
2	1.5	41	0.29	285	1.97
average			0.29±0.13		2.90±1.82

[a] Cumulative product measured in headspace.
[b] based on two independent replicates

Table S5. Optimization of photocatalytic CO_2 reduction using ZnSe-BF_4 | MEMI. Unless otherwise stated, standard conditions were: 0.5 μM QD, 0.1 M AA/$NaHCO_3$, 3 mL water under CO_2 flow (4 sccm); 100 mW cm^{-2}, AM 1.5G, λ > 400 nm, 10 h irradiation, 25 °C.

Co-catalyst	pH	n(H_2) ± σ / mmol g_{ZnSe}^{-1}	n(CO) ± σ / mmol g_{ZnSe}^{-1}	CO selectivity[a]
varying pH[b]				
ZnSe | MEMI	4.5	62.8 ± 3.14	1.77 ± 0.15	2.7% ± 0.2%
ZnSe | MEMI	5.5	50.5 ± 5.06	1.23 ± 0.06	2.4% ± 0.3%
ZnSe | MEMI	6.5	29.9 ± 8.52	1.78 ± 0.23	5.8% ± 1.1%
ZnSe | MEMI	7.5	10.7 ± 1.95	0.96 ± 0.10	8.3% ± 0.6%

[a] CO selectivity = 100 % × nCO / (nCO + nH_2).
[b] pH determined after 10 min of purging with CO_2 after it stabilized

Table S6. Computed surface energies (γ) of the XRD-predominant facets for ZnSe.

Surface facet	Area of unit cell (Å^2)	γ (J m^{-2})
(111)	57.083	1.00
(222)	57.083	1.00
(200)	32.957	1.33
(220)	46.608	0.49
(311)	109.305	0.93

Supporting Figures

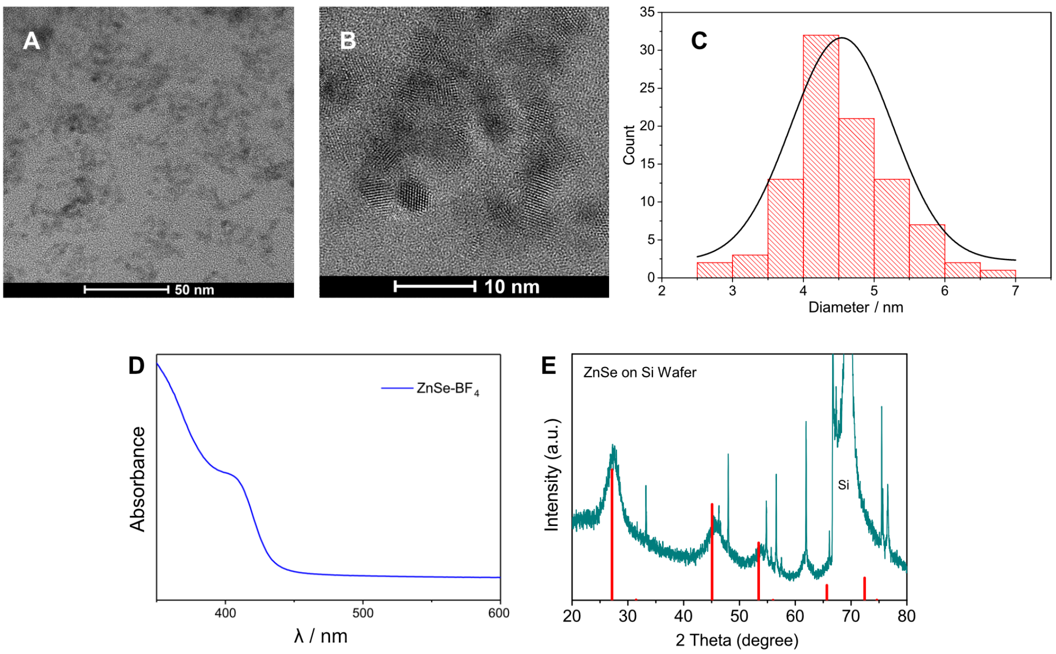

Figure S1. Characterization of ZnSe-BF_4 QDs. (A-B) Transmission electron micrographs; (C) Particle size distribution determined by TEM; (D) UV-vis absorption spectrum; (E) Powder X-ray diffractogram of ZnSe-BF_4 on Si wafer (green) overlaid with cubic zinc blende ZnSe reference (PDF 01-071-5978, red), the unassigned signals originate from the Si wafer.

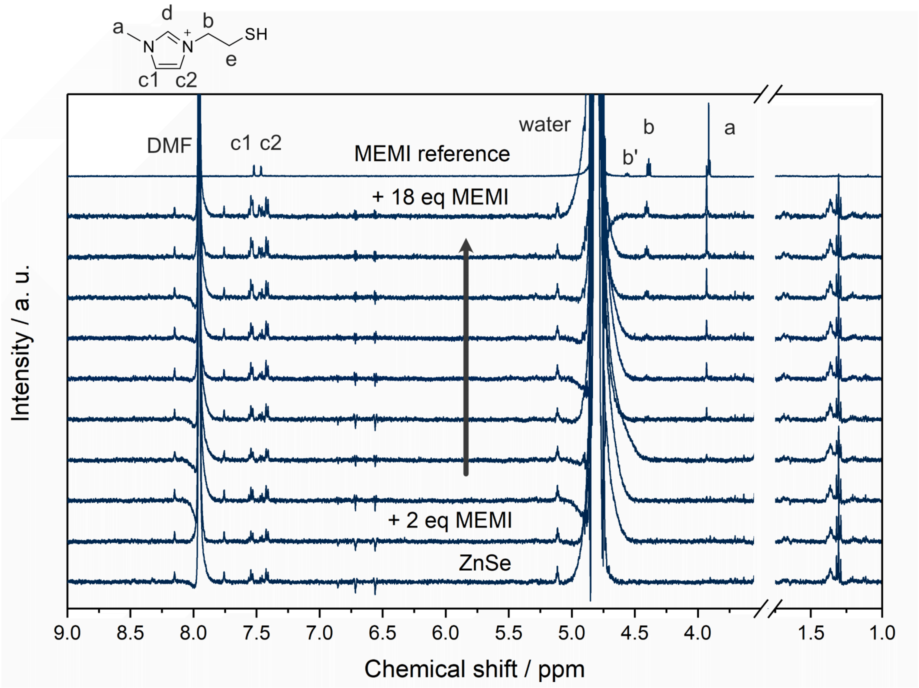

Figure S2. ^{1}H-NMR spectroscopy titration of aliquots of MEMI to a solution of 5 µM ZnSe-BF_4 QDs in D_2O. b' represents a proton of the oxidized disulfide MEMI equiv. (due to some residual oxygen present). Aromatic protons c1 and c2 are noticeable but difficult to quantify due to an overlap with an impurity in the QD stock solution. e (not shown) overlaps with an impurity from the QD stock solution (DMF). The acidic proton d is not visible (in D_2O) as seen before in the literature[22]. The region (1.7 to 3.5 ppm) was removed for clarity and contained residues of the solvents from the QD synthesis and purification (DMF, MeOH, BuOH, $CHCl_3$), for full spectrum see Figure S3. All spectra referenced to the water peak at 4.80 ppm. The sensitivity of the NMR measurement with MEMI was determined to be ~5-10 µM meaning that even the first titration (i.e. 2 *equiv.*) would be detectable by NMR spectroscopy. Small variations of the intensity of the residual water peak are caused by the NMR methodology, see Figure S3.

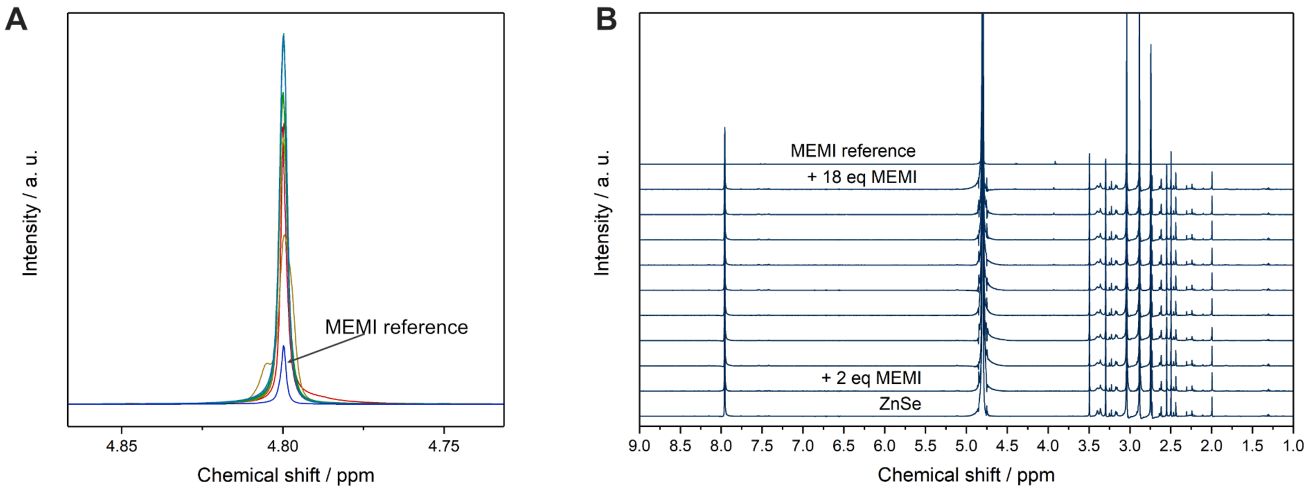

Figure S3. (A) Residual water peak of the ^{1}H-NMR spectroscopy titration of aliquots of MEMI to ZnSe-BF_4 QDs in D_2O. (B) Full spectrum of the ^{1}H-NMR spectroscopy titration.

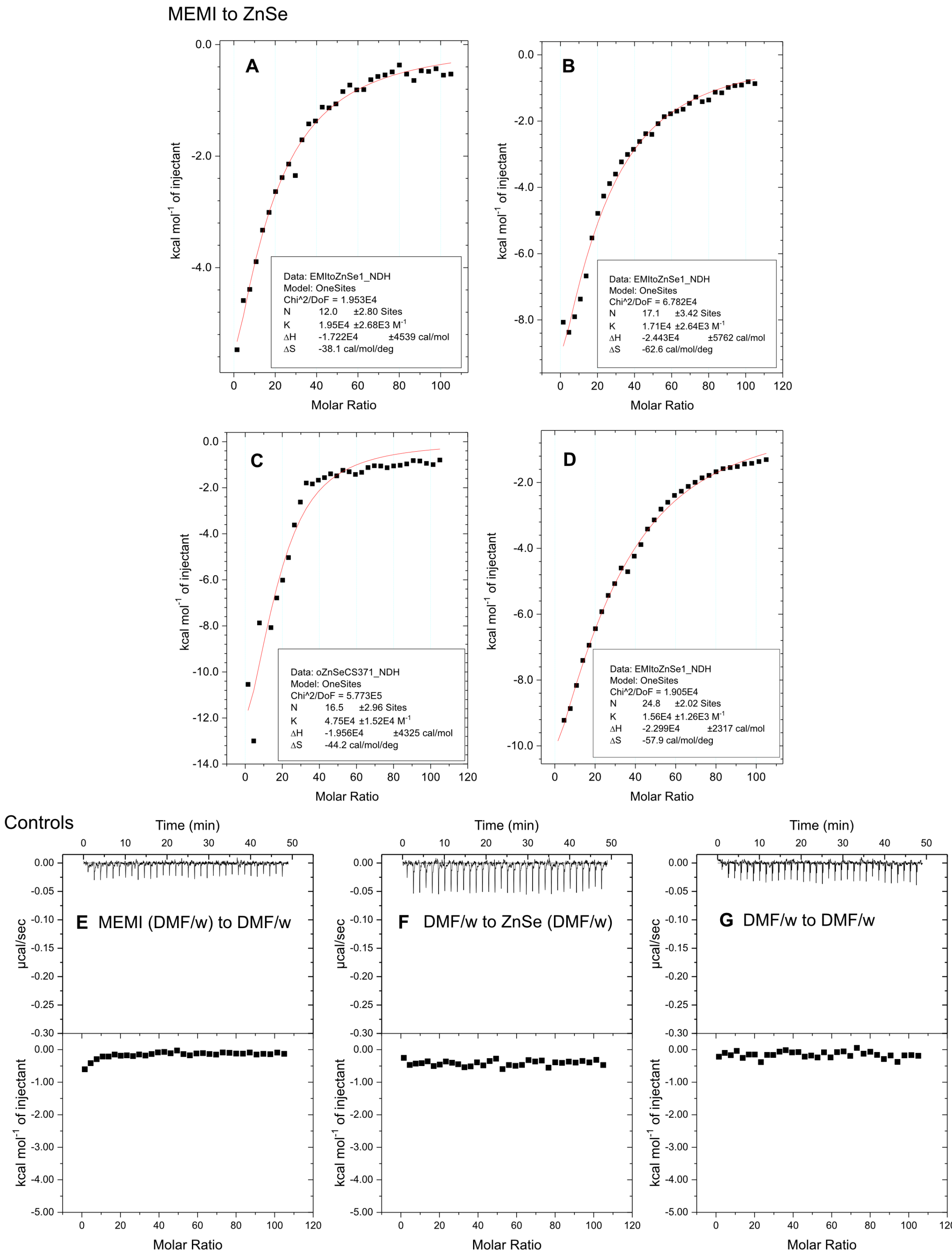

Figure S4. Supplementary ITC data. (A) – (D) ITC curves and plots for replicates of four independent titrations of MEMI (in H_2O/DMF) into ZnSe-QDs (1 μM) (A), 2 μM (B, C, D) in H_2O/DMF) (black scatter) and fit using the one set of sites binding model to estimate the number of binding sites N (red line). (E) – (G) ITC curves and plots of control experiments: (E) titration of MEMI (0.5 mM in H_2O/DMF) into H_2O/DMF, (F) H_2O/DMF into ZnSe (1 μM in DMF/H_2O) and (G) H_2O/DMF to H_2O/DMF. The DMF concentration (3.12%, v/v) was kept constant in the cell and titrant solution in all titration experiments.

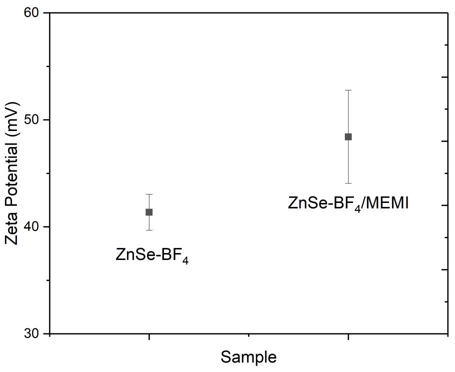

Figure S5. Electrokinetic ζ potential measurements of unfunctionalized ZnSe-BF_4 QDs and functionalized ZnSe | MEMI, in aqueous solution. Conditions: 0.5 µM ZnSe QDs, 25 µM MEMI.

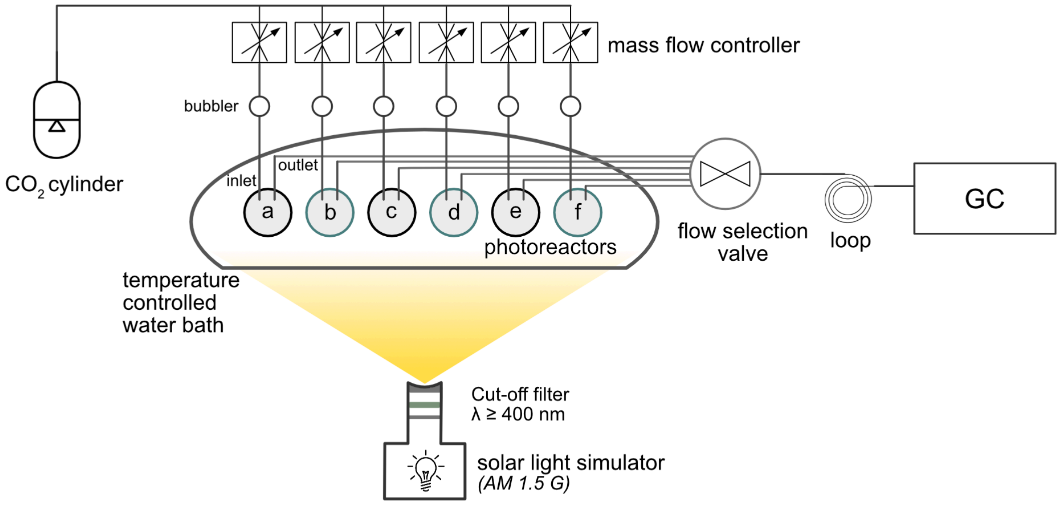

Figure S6. Schematic representation of the continuous-flow setup for photocatalytic CO_2 reduction with automated GC sampling. Samples a/c/e and b/d/f each represent one experimental condition in triplicate, evenly distributed throughout the light simulator to account for deviations in light intensity.

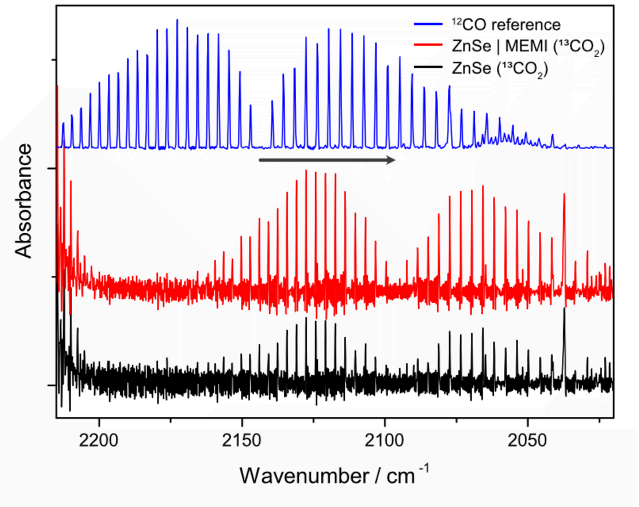

Figure S7. Isotopic labelling: Gas-phase transmission IR spectra of the CO vibration depending on the employed CO_2 isotopologue. Samples ZnSe and ZnSe | MEMI under an atmosphere of $^{13}CO_2$ compared to a reference spectrum of ^{12}CO. Conditions: AM 1.5G, λ > 400 nm, 100 mW cm^{-2}, 0.5 µM ZnSe-BF_4, 50 µM MEMI, 0.1 M AA, pH 6.5, CO_2 25 °C, 1000 min irradiation.

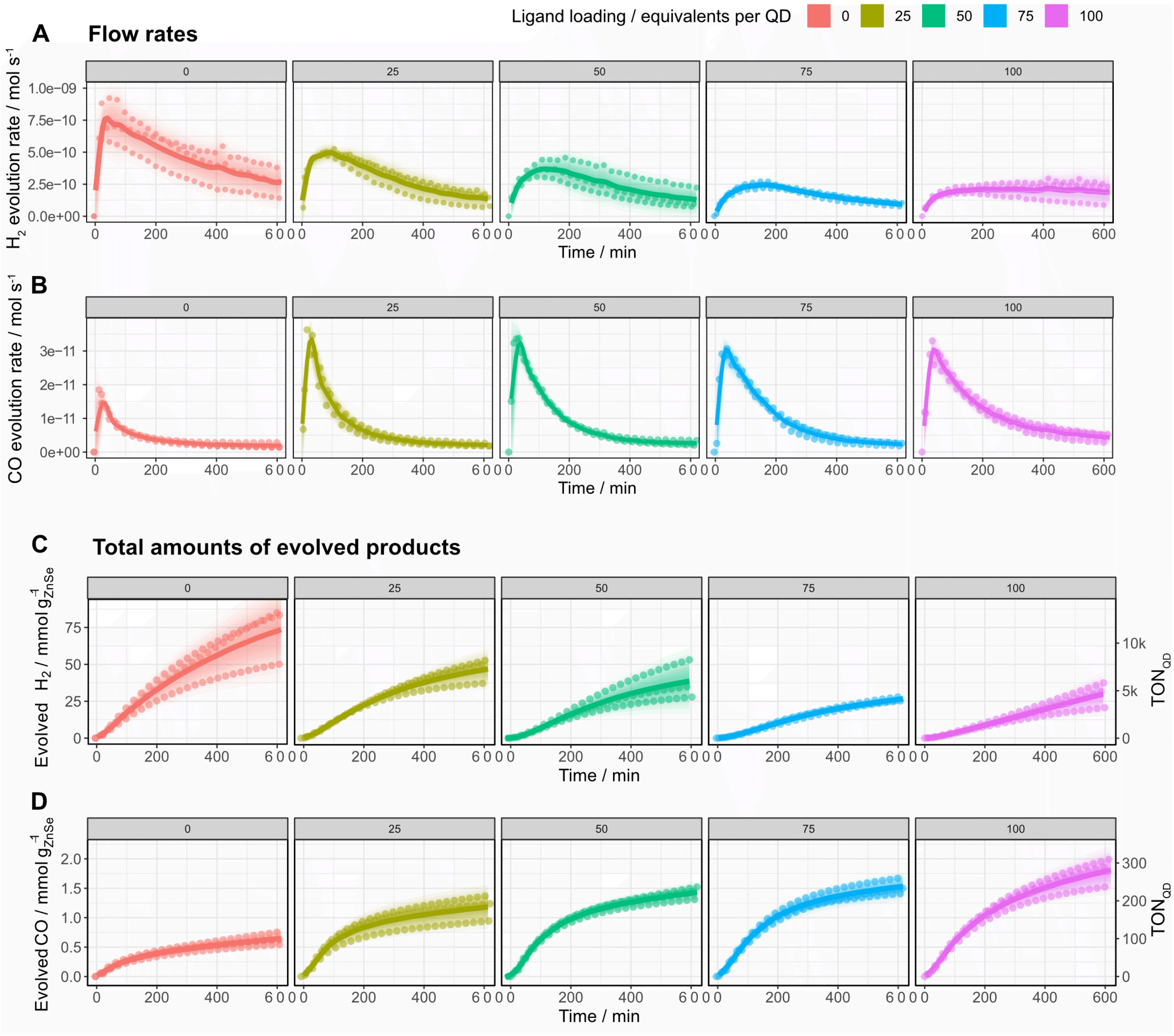

Figure S8. Photocatalytic reduction of aqueous CO_2 in the presence of ZnSe | MEMI. Influence of the ligand loading (in equiv. ligand per QD) on the product distribution: (A) H_2 and (B) CO evolution rates. (C) Total amount evolved H_2 and (D) total amount evolved CO. Conditions: AM 1.5G, $\lambda > 400$ nm, 100 mW cm^{-2}, 0.5 µM ZnSe-BF_4, 0.1 M AA/$NaHCO_3$, pH 6.5, CO_2 constant flow (4 sccm), 25°C (unless otherwise stated). The scatter plot shows measured values for a given experimental condition conducted in triplicate. The scatter plot shows measured values for a given experimental condition conducted in triplicate. Lines represent a fit of the mean of a triplicate experiment. The shaded area represents the standard deviation where the opacity is proportional to the density of the standard deviation.

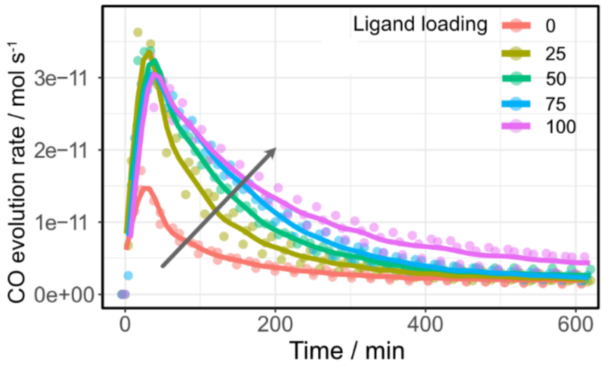

Figure S9. CO-formation rate of ZnSe-QDs in the presence of varying amounts of MEMI. Overlay of Figure S8-B for clarity. Conditions: see Figure S8.

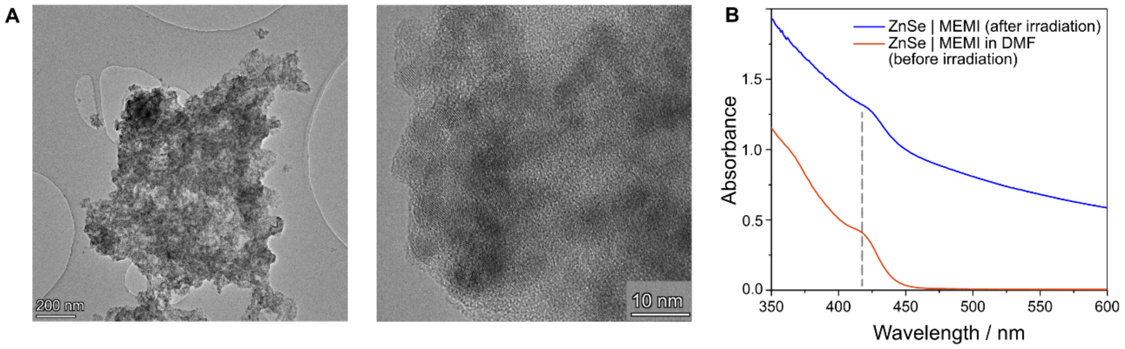

Figure S10. Post catalysis characterization. (A) Transmission electron micrographs of ZnSe-BF_4 | MEMI after irradiation (1000 min) showing aggregated structures with a nanocrystalline morphology. The particles were precipitated after photocatalysis via centrifugation. (B) UV-vis characterization of the photocatalyst after irradiation (1000 min).

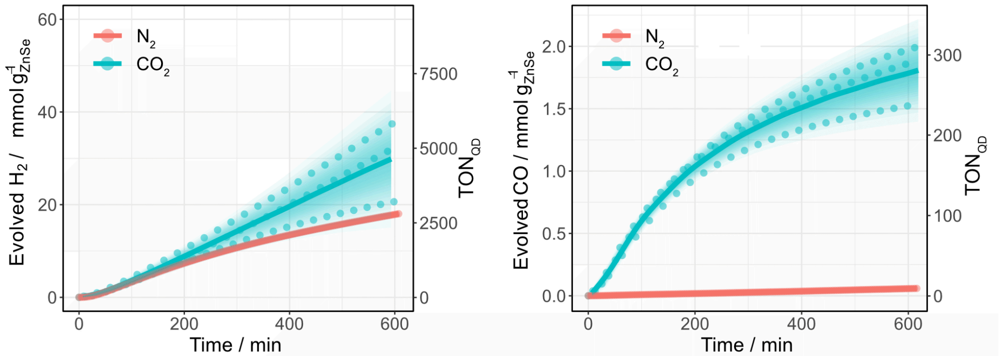

Figure S11. Photocatalytic CO_2 reduction using ZnSe | MEMI under various atmospheres. Conditions: AM 1.5G, $\lambda > 400$ nm, 100 mW cm^{-2}, 0.5 µM ZnSe-BF_4, CO_2-sample: 0.1 M AA/$NaHCO_3$, CO_2, pH 6.5; N_2-sample: 0.1 M AA pH adjusted to 6.5, constant flow (4 sccm), 25°C (unless otherwise stated).

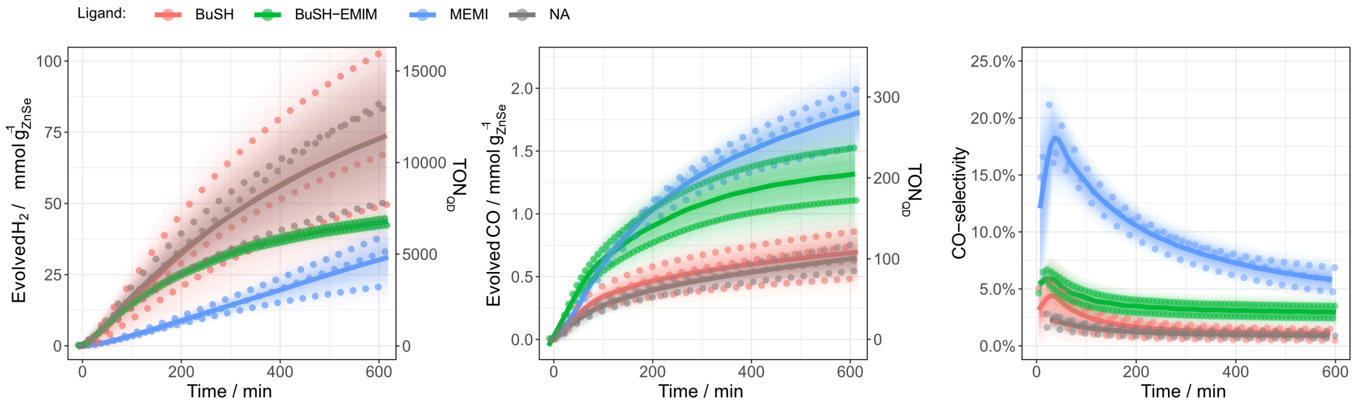

Figure S12. Benchmarking ZnSe | BuSH with ZnSe | MEMI in comparison to a mix of BuSH and EMIM-BF_4, referred to as "BuSH-EMIM" (25 µM each). Conditions: AM 1.5G, λ > 400 nm, 100 mW cm^{-2}, 0.5 µM ZnSe-BF_4, 50 µM capping ligand, 0.1 M AA/$NaHCO_3$, pH 6.5, CO_2 constant flow (4 sccm), 25°C.

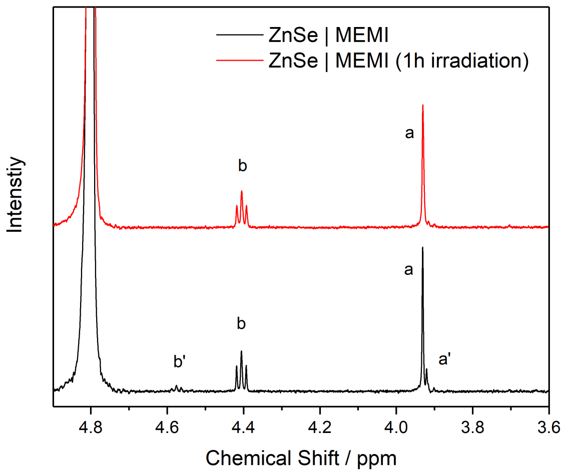

Figure S13. ^{1}H-NMR spectroscopy of ZnSe | MEMI in D_2O before and after irradiation (1µM in D_2O, CO_2, AM 1.5G, λ > 400 nm, 100 mW cm^{-2}). The spectrum before irradiation (black) exhibits a mixture of MEMI and its oxidized disulfide equiv. (due to residual O_2) indicated by signals a' and b' respectively. The disulfide signals vanish after irradiation (red) suggesting the opposite reaction, a reduction of residual disulfide rather than photocatalytic thiol oxidation.

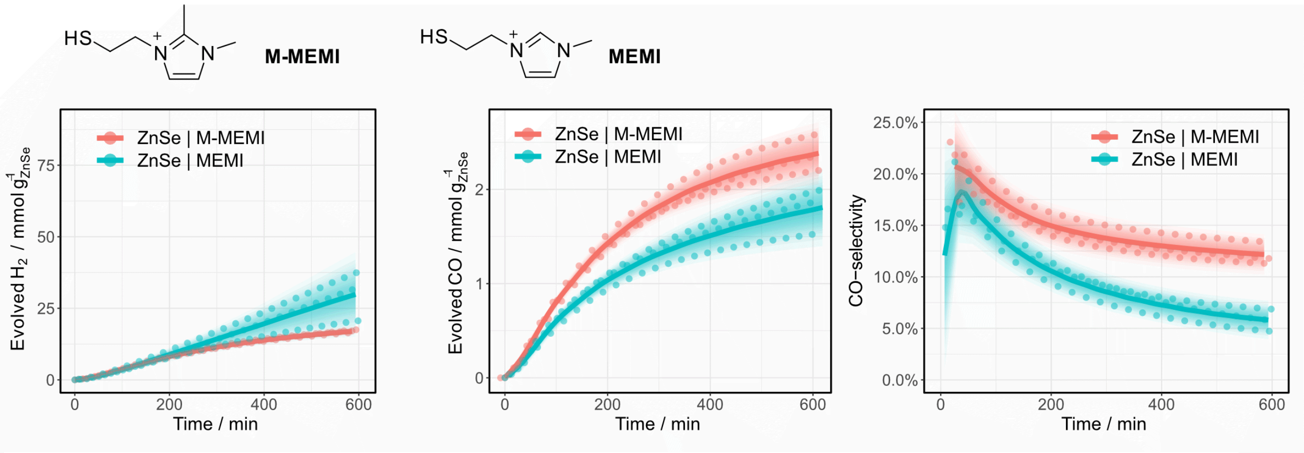

Figure S14. Benchmarking photocatalytic CO_2 reduction of ZnSe | MEMI vs. ZnSe | M-MEMI. Conditions: AM 1.5G, $\lambda > 400$ nm, 100 mW cm^{-2}, 0.5 µM ZnSe-BF_4, 50µM capping ligand, 0.1 M AA/$NaHCO_3$, pH 6.5, CO_2 constant flow (4 sccm), 25°C.

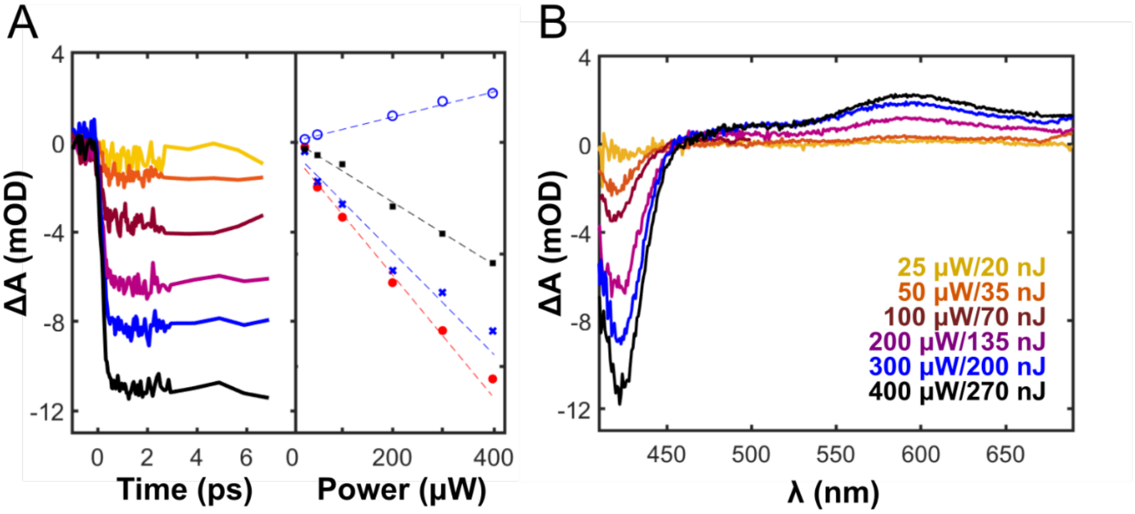

Figure S15. Transient absorption (TA) optical response at varying pump pulse energies (nJ)/power (μW) for ZnSe-BF_4 quantum dots (QDs) using a 400 nm pump. **(A)** The early-time kinetics (left column) extracted at 425 nm remain unchanged within the investigated range of pump energies, see legend of corresponding color in (B). In the rightmost column, the amplitudes (mOD) were estimated by averaging over adjacent wavelengths: 412-416 nm (red circles), 416-422 (blue crosses) nm, 435-440 nm (black squares) and 585-595 nm (blue open circles). The range from 585-595 nm corresponds to the positive transient. The signal amplitudes increase linearly within the range of investigated pulse energies. **(B)** TA spectra of ZnSe QDs at varying pump pulse energies/powers. All spectra presented in the main article are recorded with pump energies of ~70 nJ.

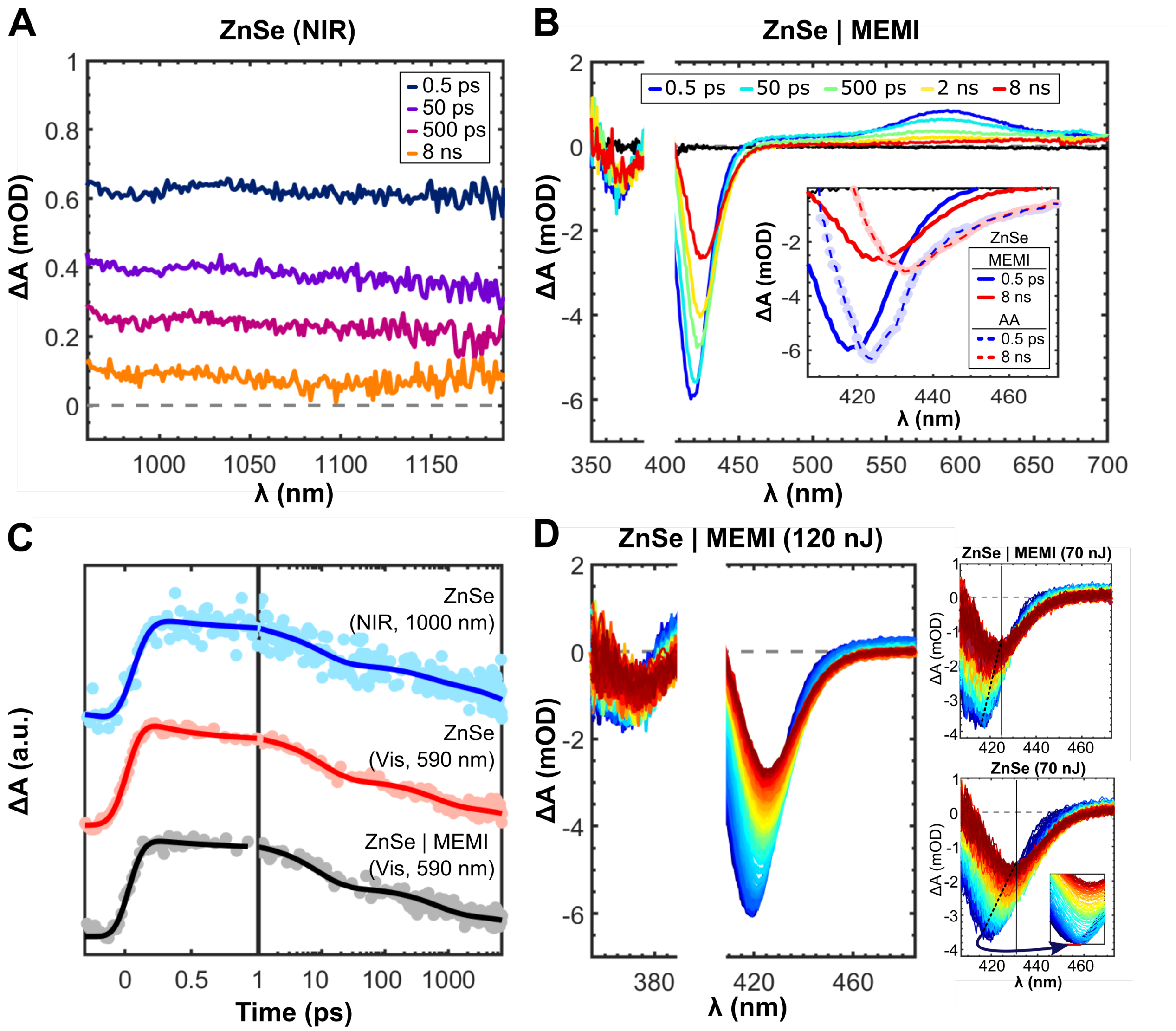

Figure S16. Transient absorption (TA) experiments using a 400 nm pump. **(A)** TA-NIR spectra of ZnSe-BF_4 QDs (170 nJ/pulse). **(B)** TA spectra of ZnSe | MEMI. Inset: Close-up of ZnSe | MEMI TA spectra compared to ZnSe | AA. The ZnSe | AA spectra show an unsymmetrical bleach band with a low-energy tail that extends to energies below the ZnSe bandgap. **(C)** TA kinetics of ZnSe-BF_4 QDs (NIR: 1000 nm, blue [6 ps, A: 0.43; 850, A: 0.35; inf, A: 0.22]; Vis: 590 nm, red [8.5 ps, A: 0.43; 830 ps, A: 0.38; inf, A: 0.19]) and ZnSe | MEMI (Vis: 590 nm, black [9.5 ps, A: 0.48; 880 ps, A: 0.36; inf, A: 0.16]). These kinetics are considered equal, within sample-to-sample variances, and reflect the population of trapped holes (see main article). **(D)** Close-up of TA spectra of ZnSe | MEMI (~120 nJ) (left), showing the spectral shift of the two bleach bands XB1 and XB2 → $XB1_T$ and XB2 $_T$. All time points between 300 fs and 8 ns are presented (blue → green → yellow → red) for the given wavelength range. Close up of TA spectra of ZnSe | MEMI at lower pump fluence (~70 nJ, top) and ZnSe (~70 nJ, bottom). The TA spectra of neat ZnSe (bottom, batch B1, see Sample Preparation) show a smaller wavelength shift with time than the spectra presented in the main article (batch B2, Figure 4A) but show a significantly larger shift than when MEMI is present (top, batch B2). The shift magnitude is indicated by dashed lines, with a starting point at ~416 nm corresponding to the center of the exciton bleach. In neat ZnSe (bottom) the bleach shifts with a few nanometers on the hundred of femtoseconds timescale, corresponding to a slight growth at ~420 nm prior to recovery (see inset from spectra recorded at higher powers).

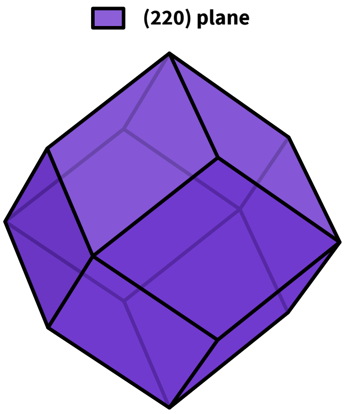

Figure S17. Wulff construction representation obtained for the ZnSe-QD using the calculated surface energies (see Table S6) of the predominant surface peaks in the experimental XRD spectra shown in Figure S1E.

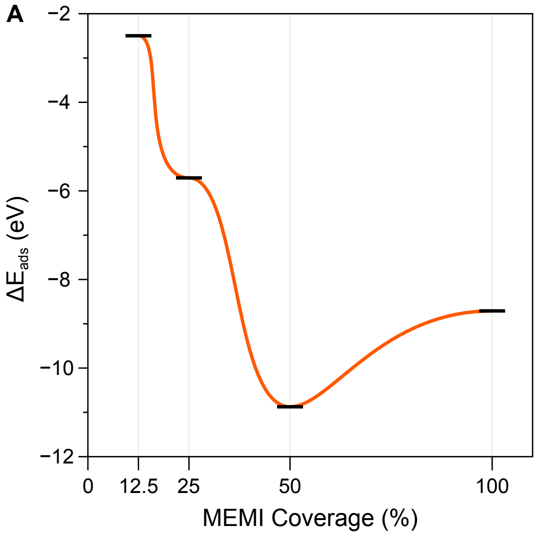

Figure S18. Coverage analysis of MEMI ligands on the ZnSe | MEMI system. A coverage of 12.5 % corresponds to a $p(2\times2)$ ZnSe(220) surface with 1 of the 8 Zn surface sites occupied by a MEMI ligand; 25 % corresponds to a $p(2\times1)$ ZnSe(220) surface with 1 of the 4 sites covered; 50 %, to a $p(2\times1)$ surface with 2 of the 4 sites covered; and 100 %, to a $p(1\times1)$ surface with all of the 2 sites covered. See Computational Methods section for details.

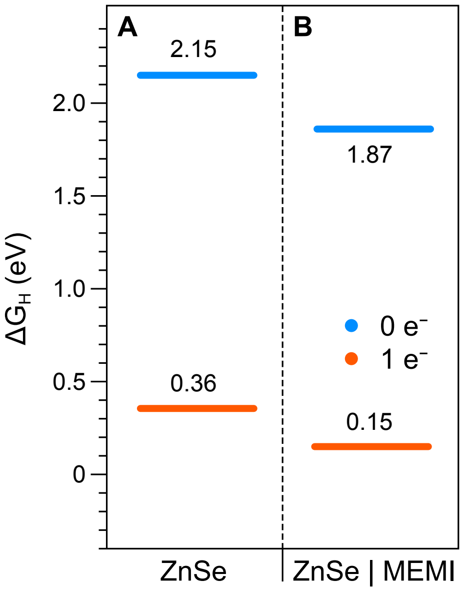

Figure S19. Gibbs adsorption energies (in eV) of a H atom, ΔG_H, atop the active surface Zn sites of a (A) bare $p(2\times1)$ ZnSe(220) surface and (B) a $p(2\times1)$ ZnSe(220) surface with a 50 % MEMI coverage. For all systems, adsorption energies have been calculated without (blue trace, 0 e^-) and with the presence of a photogenerated electron (red trace, 1 e^-) by adding an additional electron in the simulation.

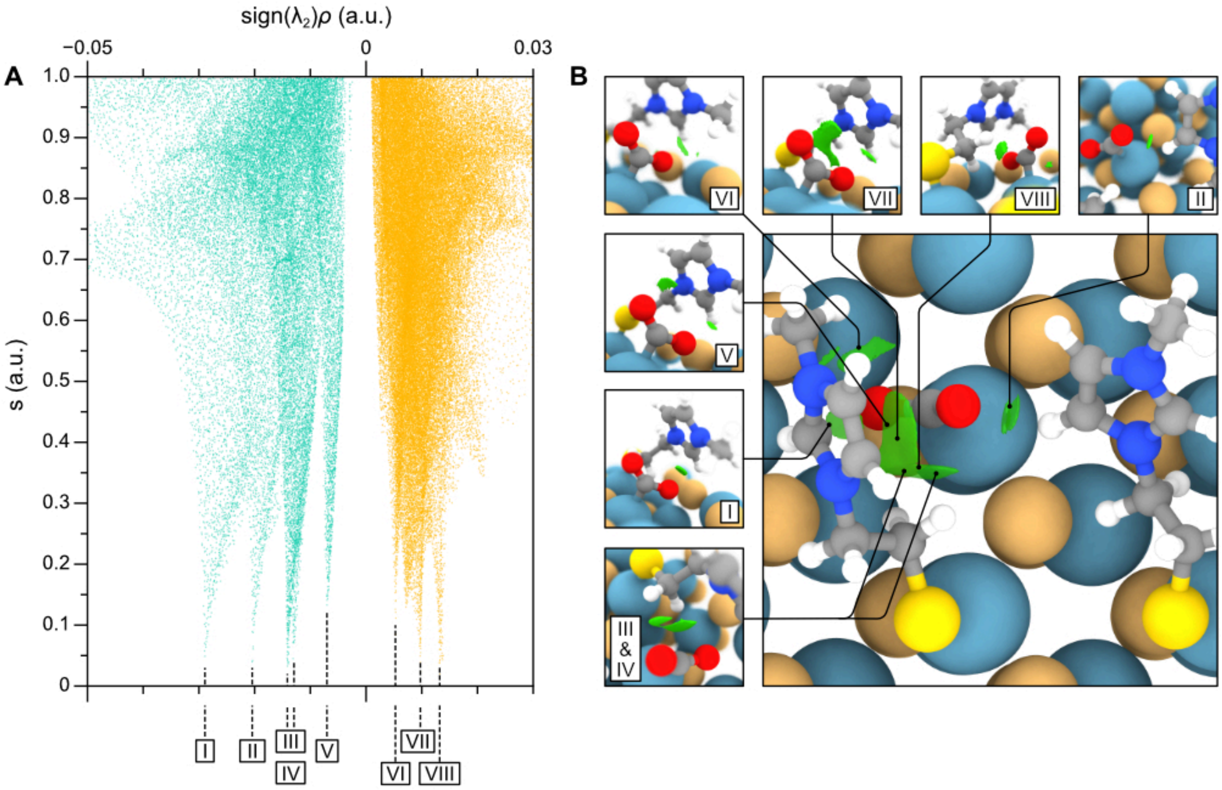

Figure S20. (**A**) Plot of the reduced density gradient (s) as a function of the electron density multiplied by the sign of the second eigenvalue of the Hessian matrix (sign(λ_2)ρ) for the ZnSe | MEMI system with the adsorbed *$CO_2^{\delta-}$ intermediate. Peaks correspond to attractive and repulsive interactions, assigned based on the value of sign(λ_2)ρ below and above 0 a.u., respectively. Thus, the further the peak is from the origin, the stronger the attractive or repulsive interaction. (**B**) Representation of the non-covalent interactions between the MEMI ligands and *$CO_2^{\delta-}$. Isosurfaces are plotted with an isovalue of 0.35 $e^-/(\text{a.u.})^3$. The separate interactions are also shown and assigned to their corresponding peaks in (A).

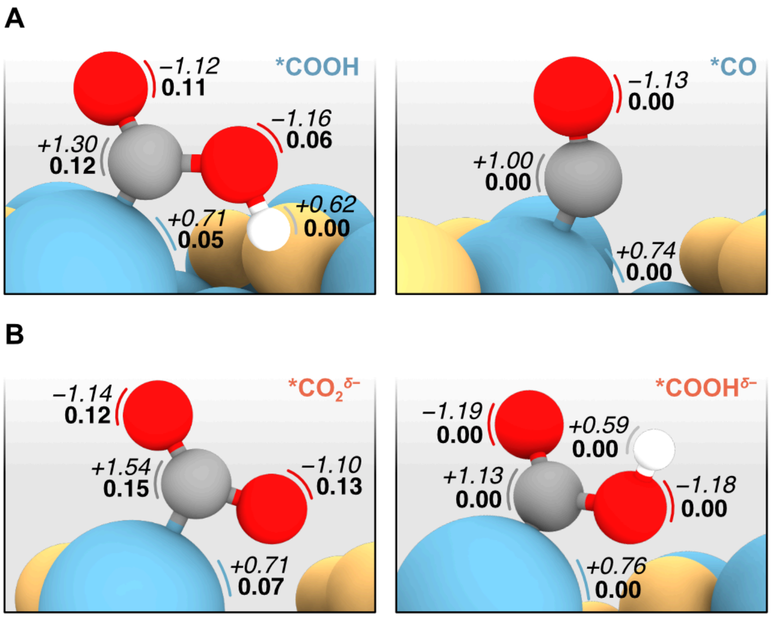

Figure S21. Side view representation of the relevant intermediates along the two proposed pathways, (**A**) the conventional mechanism where CO_2 is activated in a concerted step with a PCET forming a *COOH (Figures 5C, path *a* in the main text), and (**B**) the proposed mechanism where CO_2 interacts with a surface photogenerated electron to form $*CO_2^{\delta-}$ (Figures 5C, path *b* in the main text). Atomic Bader charges (in e) are displayed in italics beside each atom, while magnetizations (in μ_B) are shown in bold. Note that neighbouring MEMI ligands have been omitted for clarity. Colour code is the same as in Figure 5 in the main text.

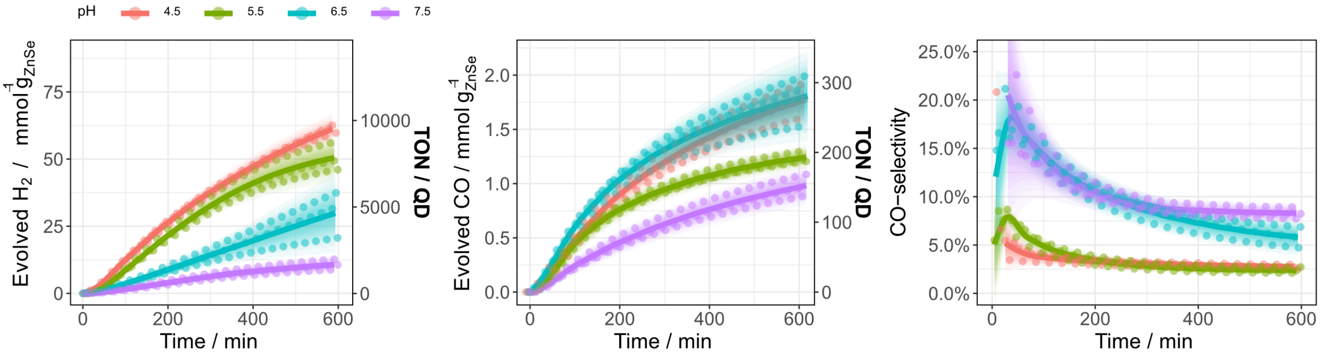

Figure S22. pH dependence of the photocatalytic CO_2 reduction using ZnSe | MEMI. Conditions: AM 1.5G, $\lambda > 400$ nm, 100 mW cm^{-2}, 0.5 µM ZnSe-BF_4, 50 µM MEMI, 0.1 M AA, 0.1 M $NaHCO_3$ (only for pH 6.5), 1 M $NaHCO_3$ (only for pH 7.5), CO_2 constant flow (4 sccm), 25°C.

Supporting References

[1] Y. Shen, R. Tan, M. Y. Gee, A. B. Greytak*, ACS Nano* **2015**, *9*, 3345–3359

[2] L. D. Field, B. A. Messerle, K. Q. Vuong, P. Turner, *Organometallics* **2005**, *24*, 4241–4250.

[3] J. Tamura, A. Ono, Y. Sugano, C. Huang, H. Nishizawa, S. Mikoshiba, *Phys. Chem. Chem. Phys.* **2015**, *17*, 26072–26078.

[4] G. I. Matiello, A. Pazini, K. I. M. da Silva, R. G. M. da Costa, G. Ebeling, J. Dupont, J. Limberger, J. D. Scholten, *Tetrahedron Lett.* **2019**, *60*, 780–784.

[5] D. Yuan, H. V. Huynh, *Dalt. Trans.* **2011**, *40*, 11698.

[6] M. Banski, M. Afzaal, M. a. Malik, A. Podhorodecki, J. Misiewicz, P. O'Brien, *Chem. Mater.* **2015**, *27*, 3797–3800.

[7] M. F. Kuehnel, D. W. Wakerley, K. L. Orchard, E. Reisner, *Angew. Chemie - Int. Ed.* **2015**, *54*, 9627–9631.

[8] E. L. Rosen, R. Buonsanti, A. Llordes, A. M. Sawvel, D. J. Milliron, B. A. Helms, *Angew. Chemie Int. Ed.* **2012**, *51*, 684–689.

[9] R Core Team. (2020).R: A language and environment for statistical computing. Vienna, Austria: R Foundation for Statistical Computing. Retrieved from https://www.R-project.org/

[10] Wickham et al., Welcome to the Tidyverse. *Journal of Open Source Software*. **2019** *4*, 1686, (https://doi.org/10.21105/joss.01686)

[11] Pav, Steven E., Documentation for Geom_cloud function. Retrieved from https://www.rdocumentation.org/packages/ggallin/versions/0.1.1/topics/geom_cloud (2020)

[12] Perdew, J. P.; Burke, K.; Ernzerhof, M. *Physical Review Letters,* **1996**, *77*, 3865–3868.

[13] Kresse, G.; Furthmüller, J. *Physical Review B*, **1996**, *54*, 11169–11186.

[14] Blöchl, P. E. *Physical Review B*, **1994**, *50*, 17953–17979.

[15] Grimme, S., Ehrlich, S. & Goerigk, L. *J. Comput. Chem.* **2011**, 32, 1456–1465.

[16] Jain, A.; Ong, S. P.; Hautier, G.; Chen, W.; Richards, W. D.; Dacek, S.; Cholia, S.; Gunter, D.; Skinner, D.; Ceder, G. *et al. APL Materials* **2013**, *1*, 011002.

[17] Tran, R., Xu, Z., Radhakrishnan, B. *et al. Sci Data* **2016**, *3*, 160080.

[18] Nørskov, J. K. *et al. J. Phys. Chem. B*, **2004**, *108*, 17886–17892.

[19] J. K. Nørskov, T. Bligaard, A. Logadottir, J. R. Kitchin, J. G. Chen, S. Pandelov, U. Stimming, *J. Electrochem. Soc.* **2005**, *152*, J23.

[20] Otero-de-la-Roza, A.; Johnson, E. R.; Luaña, V. *Comput. Phys. Commun.* **2014**, *185*, 1007−1018.

[21] Kuznetsova, V. A.; Mates-Torres, E.; Prochukhan, N.; Marcastel, M.; Purcell-Milton, F.; O'Brien, J.; Visheratina, A. K.; Martinez-Carmona, M.; Gromova, Y.; Garcia-Melchor, M.; Gun'ko, Y. K. *ACS Nano* **2019**, *13*, 13560–13572.

[22] K. I. Assaf, H. Abed alfattah, A. F. Eftaiha, S. K. Bardaweel, M. A. Alnajjar, F. A. Alsoubani, A. K. Qaroush, M. I. El-Barghouthi, W. M. Nau, *Org. Biomol. Chem.* **2020**, *18*, 2120–2128.

End of Supporting Information
